# Phlebotomine sand fly survey in the focus of leishmaniasis in Madrid, Spain (2012–2014): seasonal dynamics, *Leishmania infantum* infection rates and blood meal preferences

**DOI:** 10.1186/s13071-017-2309-z

**Published:** 2017-08-01

**Authors:** Estela González, Maribel Jiménez, Sonia Hernández, Inés Martín-Martín, Ricardo Molina

**Affiliations:** Laboratorio de Entomología Médica, Servicio de Parasitología, Centro Nacional de Microbiología, Instituto de Salud Carlos III, Ctra. Majadahonda-Pozuelo s/n, 28220 Majadahonda, Madrid Spain

**Keywords:** *Phlebotomus perniciosus*, *Leishmania infantum*, Human leishmaniasis, PCR, Blood meal preferences, Sand fly infection rates, Southwestern Madrid, Central Spain

## Abstract

**Background:**

An unusual increase of human leishmaniasis cases due to *Leishmania infantum* is occurring in an urban area of southwestern Madrid, Spain, since 2010. Entomological surveys have shown that *Phlebotomus perniciosus* is the only potential vector. Direct xenodiagnosis in hares (*Lepus granatensis*) and rabbits (*Oryctolagus cuniculus*) collected in the focus area proved that they can transmit parasites to colonized *P. perniciosus*. Isolates were characterized as *L. infantum*. The aim of the present work was to conduct a comprehensive study of sand flies in the outbreak area, with special emphasis on *P. perniciosus.*

**Methods:**

Entomological surveys were done from June to October 2012–2014 in 4 stations located close to the affected area. Twenty sticky traps (ST) and two CDC light traps (LT) were monthly placed during two consecutive days in every station. LT were replaced every morning. Sand fly infection rates were determined by dissecting females collected with LT. Molecular procedures applied to study blood meal preferences and to detect *L. infantum* were performed for a better understanding of the epidemiology of the outbreak.

**Results:**

A total of 45,127 specimens belonging to 4 sand fly species were collected: *P. perniciosus* (75.34%), *Sergentomyia minuta* (24.65%), *Phlebotomus sergenti* (0.005%) and *Phlebotomus papatasi* (0.005%)*.* No *Phlebotomus ariasi* were captured. From 3203 *P. perniciosus* female dissected, 117 were infected with flagellates (3.7%). Furthermore, 13.31% and 7.78% of blood-fed and unfed female sand flies, respectively, were found infected with *L. infantum* by PCR. The highest rates of infected *P. perniciosus* were detected at the end of the transmission periods. Regarding to blood meal preferences, hares and rabbits were preferred, although human, cat and dog blood were also found.

**Conclusions:**

This entomological study highlights the exceptional nature of the *Leishmania* outbreak occurring in southwestern Madrid, Spain. It is confirmed that *P. perniciosus* is the only vector in the affected area, with high densities and infection rates. Rabbits and hares were the main blood meal sources of this species. These results reinforce the need for an extensive and permanent surveillance in this region, and others of similar characteristics, in order to control the vector and regulate the populations of wild reservoirs.

**Electronic supplementary material:**

The online version of this article (doi:10.1186/s13071-017-2309-z) contains supplementary material, which is available to authorized users.

## Background

Leishmaniasis is caused in Spain by the trypanosomatid *Leishmania infantum* and visceral, cutaneous and mucosal forms of the disease are notified in the country, although these last two are underestimated because they are usually unnoticed [1–3]. Phlebotomine sand flies are the cornerstone in the transmission of leishmaniasis in many geographical regions of the world including the countries of the Mediterranean basin. The deep knowledge of wild cycles of *Leishmania* is still scarce despite being a very important aspect in the epidemiology of the disease. The proven vectors implicated in the transmission are *Phlebotomus perniciosus* and *Phlebotomus ariasi* [4]. Most of cases of leishmaniasis are reported in the Mediterranean coast and in central region of Spain [2]. During the period 2000–2009, 12–25 annual cases were reported in the region of Madrid (central Spain), but since 2010 an unusual increase of both visceral and cutaneous leishmaniasis cases was observed in southwestern Madrid region, mainly in four urban areas: Fuenlabrada, Leganés, Getafe and Humanes de Madrid [5, 6]. A total of 691 human cases were reported in this area between 2010 and October 2016, Fuenlabrada being the most affected town by far, where the mean incidence reached 45.17 cases/100,000 inhabitants (data provided by the Community of Madrid).

The periurban area close to the focus was traditionally used for agricultural purposes with a significant presence of hares (*Lepus granatensis*) [7]. Recently, it was modified in order to create a large green park for the enjoyment of the population living in the area. This change in land use has led to an increase of both hare and wild rabbit (*Oryctolagus cuniculus*) populations in this open space and has probably increased the population densities of sand fly vectors. Studies carried out in this leishmaniasis focus using direct xenodiagnosis have involved hares and rabbits from the area as wild reservoirs of the disease since they were able to transmit the parasite to colonized *P. perniciosus* sand flies [8, 9]. Even more, it has been demonstrated a high exposure of these lagomorphs to *P. perniciosus* bites [10]. On the other hand, high levels of leishmaniasis seroprevalence have been detected in hares and rabbits from this area [11]. These findings suggest that a sylvatic cycle of transmission of *Leishmania* exists in this periurban park independent of the classical urban domestic cycle with dog as the main reservoir [8]. Additional studies carried out in Madrid and in other regions of Spain strengthen the implication of lagomorphs in the sylvatic cycle of *L. infantum* in the country [12–14].

On the other hand, several factors associated to human activity and climate change are influencing *Leishmania* distribution in some parts of the world [15]. Studies have reported changes in sand fly distribution, as well as risk for their establishment in new areas of Europe [16]. Specifically, in the Iberian Peninsula epidemiological and entomological studies have been performed in order to update and improve the knowledge of the eco-epidemiology of leishmaniasis and the distribution of its vectors [17–20]. In most of these studies entomological surveys when combined with molecular procedures demonstrate to be worthwhile in the understanding of the epidemiology of leishmaniasis.

This work aimed to carry out a detailed entomological survey in order to obtain information about sand fly seasonal trends and densities, the evolution of sand fly infection rates, and their blood meal preferences in the exceptional human focus of leishmaniasis that is affecting urban areas of the southwestern Madrid region (Spain).

## Methods

### Study area

**Fig. 1:**
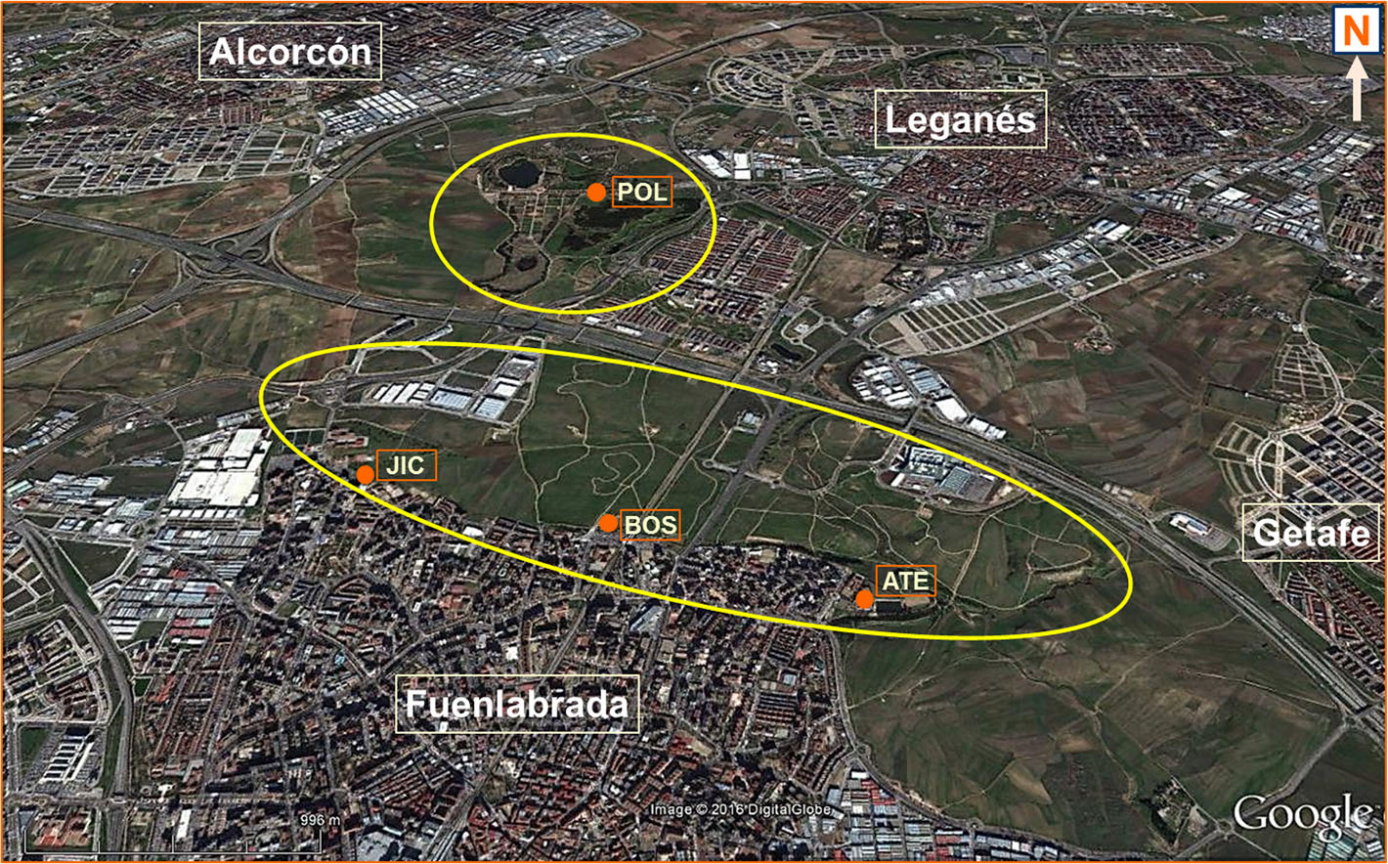
Map of the area of study in southwestern region of Madrid (Spain) showing the location of the four stations selected

The entomological surveys were conducted in a periurban green park of around 450 ha surrounded by the towns of Fuenlabrada, Leganés and Getafe (Madrid, Spain), the area affected by the leishmaniasis outbreak (Fig. 1). Three stations located in the bordering zone of the park with the urban area of the town of Fuenlabrada were chosen for the study, named ATE (40.292849N, 3.780539W), BOS (40.298084N, 3.793136W) and JIC (40.299954N, 3.806019W). A fourth station located inside the green park belonging to the municipality of Leganés was also selected, named POL (40.324903N, 3.796381W) (Fig. 1). These four stations were selected according to the high number of sand flies collected in a preliminary survey performed in 2011. The altitude of these stations varies from 655 to 691 m. The studied area belongs to the meso-Mediterranean bioclimatic zone, with an annual average temperature of 15 °C and annual precipitation of 365 mm (data from the Spanish Meteorological Agency). The characteristics of the soil for traditional rainfed cultivation make the area an exceptional habitat for hare and rabbit. The stations JIC, BOS and ATE are located in the contact line between the urban area and the green park where rabbits are abundant. In contrast, POL station is located in the middle of the green park where hares are the predominant leporids. Occasional colonies of stray cats can also be seen.

### Sand fly collection and identification

Sand flies were collected every month for three successive years (2012, 2013 and 2014) in the active season of sand flies, from June to October. Twenty sticky traps (ST) (20 × 20 cm paper sheets soaked in castor oil) and two CDC miniature light traps (LT) were used during two consecutive nights in each station. LT were replaced every morning (Fig. 1). Temperature and relative humidity (RH) were registered every 10 min using data loggers (Velleman® DVM171HD, Gavere, Belgium) hanged on each LT, with the exception of June 2012 in which the data were obtained from the nearest meteorological station located in Getafe. For phenology studies mean temperature and RH registered from dusk to dawn were used, according to information provided by the National Astronomical Observatory (Madrid).

Sand flies collected by ST were detached from the paper sheets using a fine brush, placed in 96% ethanol (to remove the castor oil) and then stored in 70% ethanol at 4 °C until processing. Female sand flies collected with LT were immediately dissected. Non-dissected females as a result of the high number of catches obtained in some cases and all males were stored in 70% ethanol at 4 °C. Blood-fed sand flies from both LT and ST were separated and stored in 70% ethanol at 4 °C for subsequent molecular studies.

Taxonomical identification of the collected sand flies was based on the morphology of the male genitalia and spermathecae of females according to Gil-Collado et al. [21]. Female genitalia was cleared in Marc-André medium and transferred together with the head to a drop of Hoyer’s mounting medium on a glass slide for identification under a microscope.

### Isolation of promastigotes from infected sand flies and further characterization

#### Isolation method

Alive female sand flies collected with LT were anesthetized with CO_2_ and placed in a sterile Petri dish containing 5% detergent solution in sterile PBS. They were then individually transferred to a drop of sterile PBS placed on a sterilized glass slide. Sand flies were dissected by using flame-disinfected entomological needles and examined for the presence of promastigotes under a phase-contrast microscope [22]. Promastigotes from guts of infected sand flies were transferred to screw crap tubes containing 200 μl of M199 medium supplemented with 20% fetal calf serum (FCS) (Hyclone™, GE Healthcare Life Science, Logan, Utah, USA) and 1.6% penicillin-streptomycin (10,000 U/ml of each antibiotic), pH 7.4 (Lonza BioWitthaker®, Verviers, Belgium). After 3–4 days at 27 °C positive vials containing live promastigotes were transferred to Novy-MacNeal-Nicolle (NNN) medium with RPMI medium for mass growth of promastigotes and further characterization of the isolates. Finally, after 1–4 weeks the promastigote cultures were stored in liquid nitrogen, depending on the evolution of their growth.

#### DNA extraction

Sand flies preserved in 70% ethanol were washed individually in sterile distilled water placed in ELISA microtiter plates. Afterwards, head, wings, genitalia, and legs of each female sand fly were removed. The genitalia and the head were processed for taxonomical identification as previously described. The presence of a blood meal was determined by observation under a stereomicroscope. The phase of blood digestion was determined according to Dolmatova & Demina [23]. Thorax and abdomen of each sand fly were used for DNA extraction using the DNeasy® Blood & Tissue Extraction Kit (Qiagen, Hilden, Germany) according to the manufacturer’s instructions. DNA from promastigote cultures was obtained by using the same kit. In both cases, two DNA elutions of 100 μl were obtained and further quantification and purity were determined by spectrophotometry with a NanoDrop ND-1000 spectrophotometer (Nanodrop Technologies, Wilmington, DE, USA). Finally, samples were stored at -20 °C until use.

#### Molecular characterization

Molecular characterization of the isolates was carried out by amplification of internal transcribed spacer regions 1 (ITS1) and 2 (ITS2) using the two pairs of primers as described in previous studies [9, 24]: (i) LITSR (5′-CTG GAT CAT TTT CCG ATG-3′) and L5.8S (5′-TGA TAC CAC TTA TCG CAC TT-3′) and (ii) L5.8SR (5′-AAG TGC GAT AAG TGG TA-3′) and LVTSV (5′-ACA CTC AGG TCT GTA AAC-3′). PCR products were separated on 1.5% agarose gel (Conda, Torrejón de Ardoz, Spain) stained with “Pronasafe Nucleic Acid Staining Solution” (10 mg/ml) (Conda, Torrejón de Ardoz, Spain) and visualized under UV light. Bands obtained were removed from the gel under UV exposure and purified with the QIAquick® Gel Extraction Kit (Qiagen). Afterwards, the DNA samples were sequenced with ABI PRISM 3730XL DNA Analyzer (Applied Biosystems, Foster City, CA, USA). Electropherograms were manually inspected and corrected using ChromasPro program (McCarthy, Queensland, Australia). Nucleotide sequences obtained were analysed with DNASTAR program (Lasergen v7.1®, Madison, WI, USA). Homologies with the available sequences data in GenBank was carried out with the software BLAST (http://www.ncbi.nlm.nih.gov/BLAST).

### Blood-feeding preferences of sand flies

Blood meal identification of blood-fed sand flies was conducted by the amplification of a fragment of 359 bp of vertebrate cytochrome *b* (*cyt b*) gene followed by sequencing as described before [9, 24]. Universal primers cyto 1 (5′-CCA TCA AAC ATC TCA GCA TGA AA-3′) and cyto 2 (5′-CCC CTC AGA ATG ATA TTT GTC CTC-3′) [25] were used. Degenerated primers cyt_bb1 (5′-CCA TCM AAC ATY TCA DCA TGA TGA AA-3′) and cyt_bb2 (5′-GCH CCT CAG AAT GAY ATT TGK CCT CA-3′) were used in samples unsuccessfully amplified with non-degenerated primers [26]. In the case of *Sergentomyia minuta* blood meal identification, degenerated primer for reptiles (5′-GCH GAY ACH WVH HYH GCH TTY TCH TC-3′) combined with primer cyto 2 was used [27]. Band purification and subsequent sequencing were carried out as previously described.

### Molecular detection of *Leishmania* DNA in sand flies

Detection of *Leishmania* was carried out by amplification of a 120 bp fragment from kDNA and further confirmation by cysteine proteinase *b* (*cpb*)-PCR following previous protocols [24]. DNA obtained from sand flies reared in the laboratory was used as negative control. DNA of *L. infantum* was used as a positive control. To prevent PCR contamination, sample preparation, reactions set-up, and PCR amplifications were performed in separate rooms, using different lab coats and gloves.

### Data analysis

Relative abundance (RA) of each species was assessed by using LT (sand flies captured of one specific species/total sand flies captured by LT), while density was calculated by using sand flies collected by ST (number of sand flies captured per m^2^ of recovered ST). Sex ratio was calculated as the number of males/females × 100.

Statistical analysis with Kruskal-Wallis test was performed in order to study significant differences in sand fly captures, density, RA, temperature and RH between the three survey periods. For the analysis of bioclimatic parameters in June, Mann-Whitney test was performed in order to compare 2013 and 2014 data. Dunn’s multiple comparison tests was used to analyse differences between mean values of the mentioned parameters between each period and month. The possible relation between sand fly captures during the three periods and bioclimatic parameters (mean/ maximum/minimum temperature and RH) was determined using the Spearman’s correlation test. Analyses were only performed with *P. perniciosus* and *S. minuta* data.

## Results

### Sand fly identification and general data

**Fig. 2:**
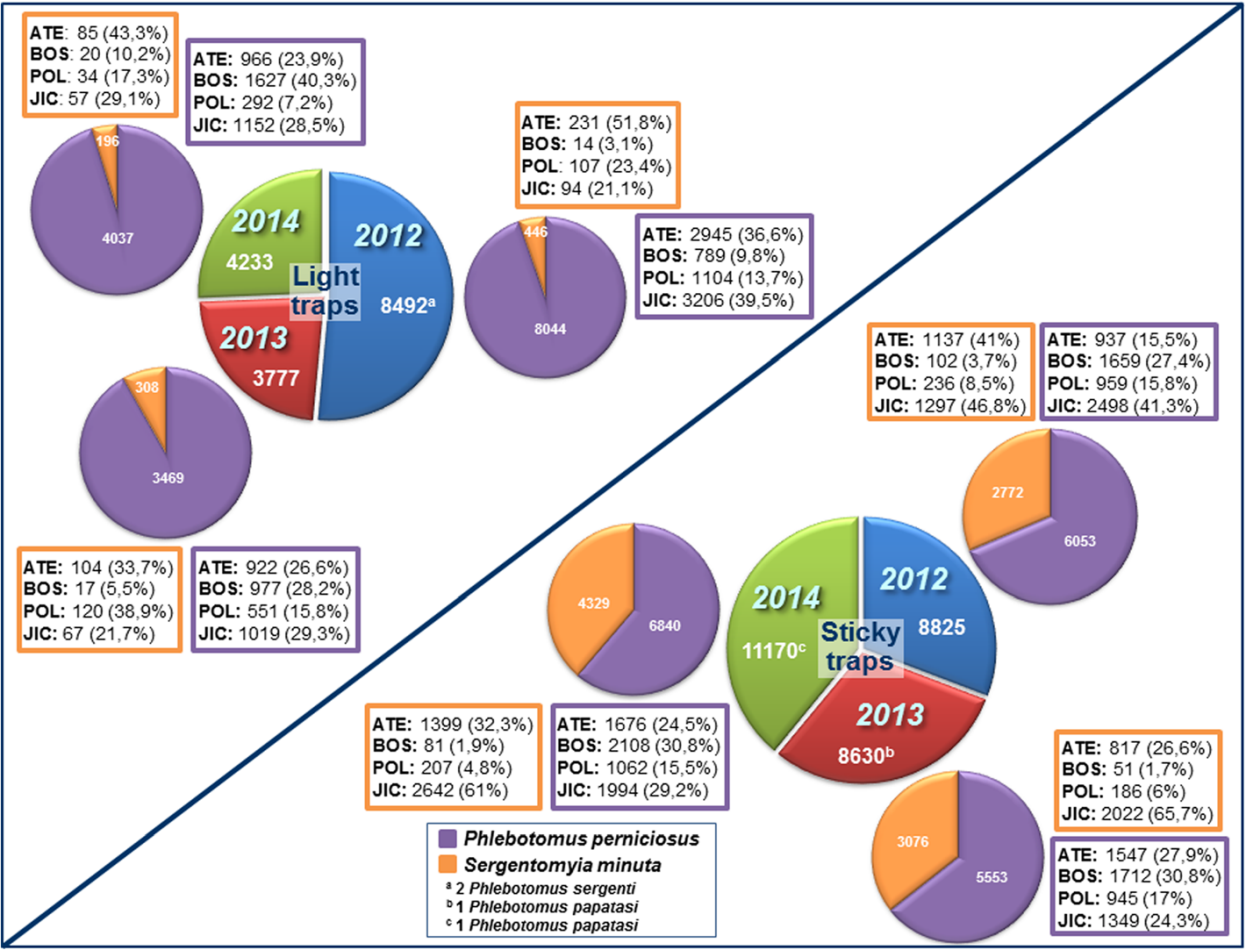
Sand flies collected at each station during the three survey periods by light traps and sticky traps. ^a^Two female *Phlebotomus sergenti* collected, one at JIC in July and one at ATE in August; ^b^One male *Phlebotomus papatasi* collected at BOS in July; ^c^One female *P. papatasi* collected at ATE in June

A total of 45,127 specimens were collected during the three survey periods: 2012 (*n* = 17,317); 2013 (*n* = 12,407); and 2014 (*n* = 15,403). A total of 16,502 sand flies were captured by LT and 28,625 were captured by ST. The two predominant species captured were *P. perniciosus* and *S. minuta*. There were no statistically significant differences between LT captures among the three periods (Kruskal-Wallis H-test: *χ* ^2^ = 5.9915, *df* = 2, *P* = 0.3833 for *P. perniciosus*, *P* = 0.7591 for *S. minuta*). No significant differences were found between *P. perniciosus* and *S. minuta* captured by ST among the three surveys, *P* = 0.4441, *P* = 0.5824, respectively. Concerning the four stations studied, the highest number of sand flies was found in JIC (*n* = 17,398), followed by ATE (*n* = 12,768), BOS (*n* = 9158) and POL (*n* = 5803). The same correlation was separately observed in the collections obtained with both LT and ST, although numbers and species of sand fly captured by each method was very variable depending on the station as shown in Fig. 2. Statistical analysis through the Kruskal-Wallis test showed no significant differences in *P. perniciosus* collections between the four stations with both capture traps (Kruskal-Wallis H-test: *χ* ^2^ = 7.8147, *df* = 3, *P* = 0.333, *P* = 0.0877 for LT and ST, respectively). However, *S. minuta* captures showed significant differences (Kruskal-Wallis H-test, *χ* ^2^ = 7.8147, *df* = 3; *P* = 0.0328 and *P* = 0.0003 for LT and ST, respectively). Dunn’s multiple comparison test showed significant differences in ST captures between stations BOS and JIC (*P* = 0.0195).

Four sand fly species were identified: *P. perniciosus* (*n* = 33,996; 75.34%), *Phlebotomus sergenti* (*n* = 2; 0.005%), *Phlebotomus papatasi* (*n* = 2; 0.005%) and *S. minuta* (*n* = 11,127; 24.65%). No *P. ariasi* was found. *Phlebotomus sergenti* (*n* = 2) were captured by LT in 2012 (1 in August-ATE and 1 in July-JIC). *Phlebotomus papatasi* (*n* = 2) were collected by ST in 2013 (July-BOS) and in 2014 (June-ATE). Mean density of *P. perniciosus* captured with ST in the three surveys was 193.6 specimens/m^2^. During the three surveys mean densities were 190.1, 175.72 and 220.45 specimens/m^2^ in 2012, 2013 and 2014, respectively. In the case of *S. minuta* the mean density was 106.81 specimens/m^2^, with variation between the surveys (87.06 in 2012; 97.34 in 2013 and 135.96 in 2014). Mean density of *P. papatasi* was 0.021 specimens/m^2^. Density values of *P. perniciosus* and *S. minuta* did not show significant variation between the three surveys (Kruskal-Wallis H-test: *χ* ^2^ = 5.9915, *df* = 2, *P* = 0.4163, *P* = 0.5824, respectively).

Regarding to RA calculated from LT captures, the mean value for *P. perniciosus* was 94.24%. Variation through the three periods was 94.74%, 91.85% and 95.37% in 2012, 2013, and 2014, respectively. This variation was not significant (Kruskal-Wallis H-test: *χ* ^2^ = 5.9915, *df* = 2, *P* = 0.0853). With regard to *S. minuta*, RA mean value was 5.75%. RA fluctuation through the three periods was 5.24% in 2012, 8.15% in 2013 and 4.63% in 2014, showing no significant deviation (Kruskal-Wallis H-test: *χ* ^2^ = 5.9915, *df* = 2, *P* = 0.4441). The mean RA value for *P. sergenti* was 0.012% (Table 1).

**Table 1:** Sand fly collections, male rate, density and relative abundance

	Year	2012				2013				2014				Total				
	Sand fly species	*P. perniciosus*	*S. minuta*	*P. sergenti*	Total	*P. perniciosus*	*S. minuta*	*P. papatasi*	Total	*P. perniciosus*	*S. minuta*	*P. papatasi*	Total	*P. perniciosus*	*S. minuta*	*P. sergenti*	*P. papatasi*	Total
Sticky trap	No. of collected sand flies	6053	2772	0	8825	5553	3076	1	8630	6840	4329	1	11,170	18,446	10,177	0	2	28,625
	Male rate (%)	89.98	59.87	0	–	90.96	56.6	100	–	89.77	56.78	0	–	90.2	56.75	0	50	–
	Density (sand flies/m^2^)	190.1	87.05	0	–	175.72	97.34	0.031	–	220.45	135.96	0.031	–	193.60	106.81	0	0.021	–
Light trap	No. of collected sand flies	8044	446	2	8492	3469	308	0	3777	4037	196	0	4233	15,550	950	2	0	16,502
	Male rate (%)	46.57	68	0	–	60.1	50.32	0	–	57.4	75	0	–	53.13	63.72	0	0	–
	Relative abundance (%)	94.74	5.24	0.02	–	91.85	8.15	0	–	95.37	4.63	0	–	94.21	5.75	0.012	0	–

**Fig. 3:**
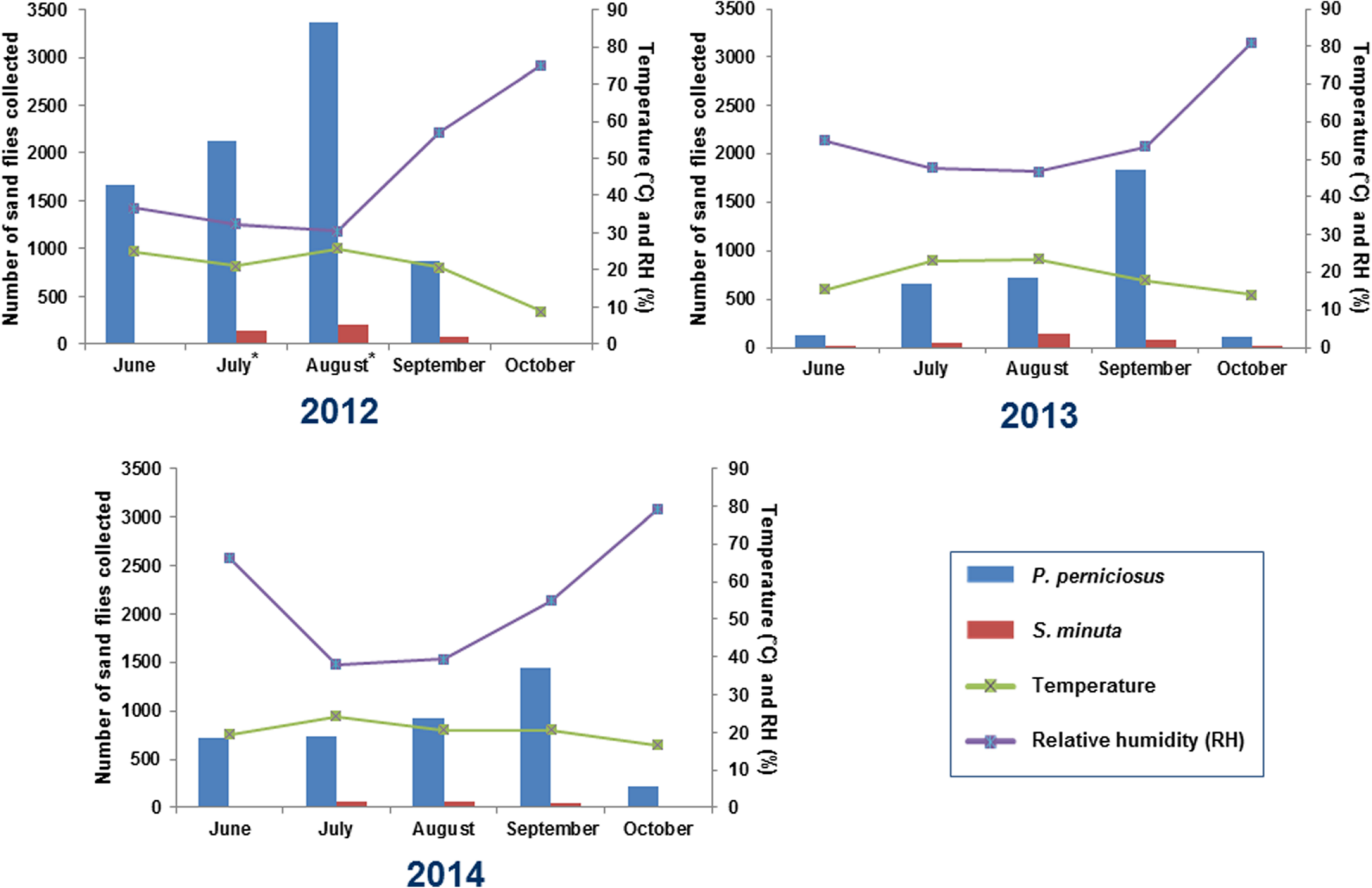
Monthly collections of *Phlebotomus perniciosus* and *Sergentomyia minuta* by light traps in 2012, 2013 and 2014 and their relation with mean temperature and relative humidity. *Two female *Phlebotomus sergenti* collected in 2012, one at JIC in July and one at ATE in August

**Fig. 4:**
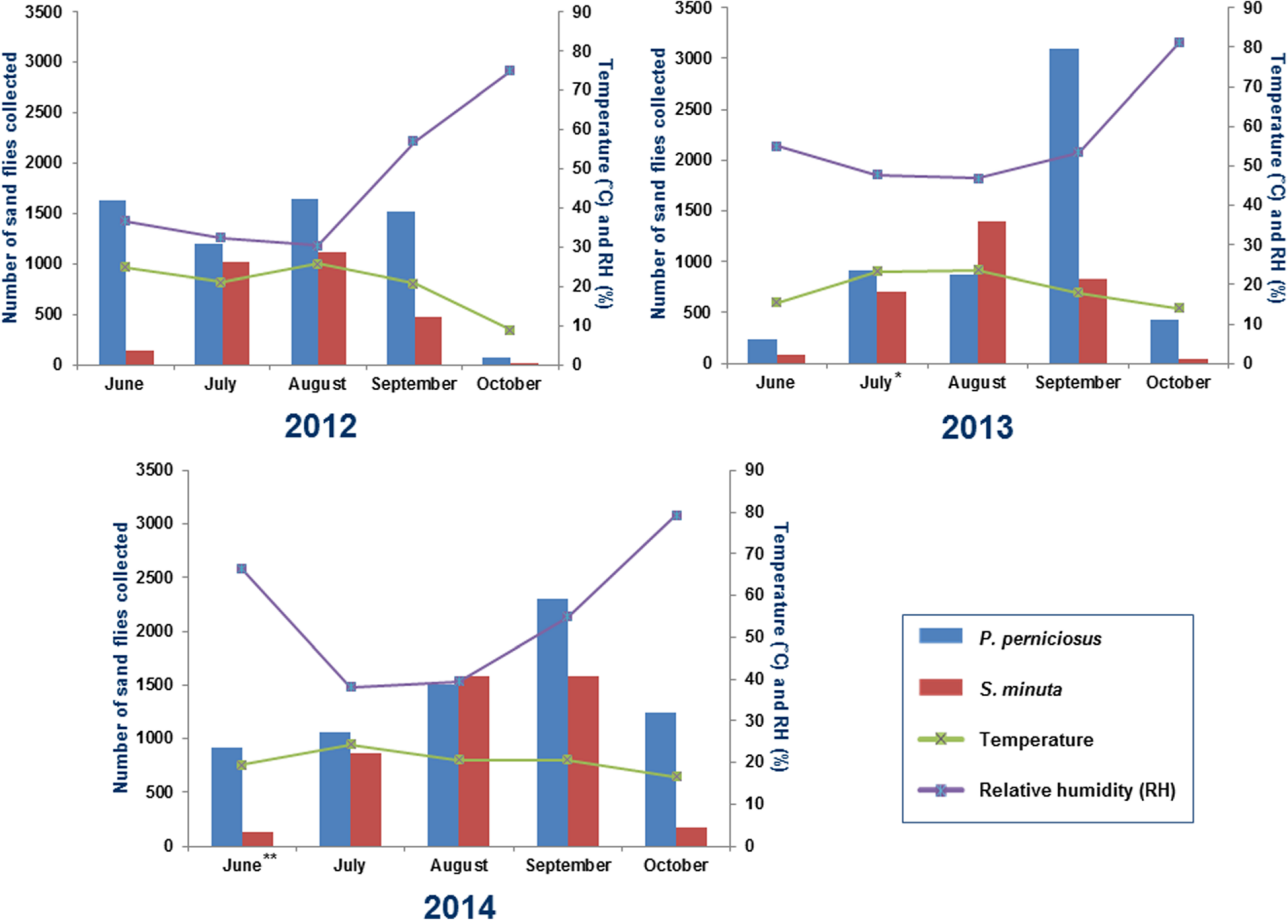
Monthly collections of *Phlebotomus perniciosus* and *Sergentomyia minuta* by sticky traps in 2012, 2013 and 2014 and their relation with mean temperature and relative humidity. *One *Phlebotomus papatasi* collected in July 2013. **One *Phlebotomus papatasi* collected in June 2014

Captures with LT showed a clear predominance of *P. perniciosus*. This species showed a peak in August 2012 and in September in 2013 and 2014 (Fig. 3). Although only significant variation was found in monthly captures of *S. minuta* during the three periods (Kruskal-Wallis H-test: *χ* ^2^ = 5.9915, *df* = 2; *P* = 0.0766, *P* = 0.0025, for *P. perniciosus* and *S. minuta*, respectively). Specifically, multiple comparison tests showed significant variation in *S. minuta* captures between August and October (*P* = 0.0467). The difference between the two predominant species was less marked with ST (Fig. 4). In any case, the number of *P. perniciosus* captured was higher than *S. minuta*, with the exception of August 2013 and 2014. *Phlebotomus perniciosus* density showed two peaks in June and August 2012, while in 2013 a weak peak in July and a higher one in September were observed. In 2014, *P. perniciosus* density only showed one peak (September). On the other hand, *S. minuta* showed a peak in August during the three periods studied; however, in September 2014 the captures also remained high (Fig. 4). Significant differences in *P. perniciosus* monthly densities between each period were found (Additional file 1: Table S1).

*Phlebotomus perniciosus* male rate exceeded 50% with both LT and ST during all surveys, except with LT in 2012 whereas *S. minuta* male rate also appeared more than 50% in ST during the three surveys periods (Table 1).

### Bioclimatic parameters variation and relation with sand fly captures

Relative humidity and temperature of this study corresponds to values recorded between dusk and dawn of the two nights of each survey. The different bioclimatic parameters studied in this work presented fluctuations between the three survey periods. On one hand, mean RH presented significant variation in June (Kruskal-Wallis H-test: *χ* ^2^ = 5.9915, *df* = 2; *P* = 0.0286). On the other hand, Dunn’s test of maximum RH showed significant differences between July 2012, 2013 (*P* = 0.0003); August 2012, 2013 (*P* = 0.0442) and August 2012, 2014 (*P* = 0.0001). Regarding minimum RH, Dunn’s test showed significant differences between August 2012, 2013 (*P* = 0.0217), August 2013, 2014 (*P* = 0.0001), October 2012, 2013 (*P* = 0.0195) and October 2012, 2014 (*P* = 0.0001) (Additional file 2: Table S2).

In case of temperature variation, no significant differences were found in monthly mean temperatures recorded over the 3 years (Additional file 3: Table S3). However, maximum temperatures showed significant fluctuations during the three collection periods except in September (Kruskal-Wallis H-test: *χ* ^2^ = 5.9915, *df* = 2; June, *P* = 0.0002; July, *P* = 0.0468; August, *P* = 0.0007; September, *P* = 0.0621; October, *P* = 0.0003). Regarding minimum temperatures significant differences were observed during the three sampling periods (Kruskal-Wallis H-test:* χ* ^2^ = 5.9915, *df* = 2; June, *P* = 0.0002; July, *P* = 0.0358; August, *P* = 0.0031; September, *P* = 0.0063; October, *P* = 0.0001) (Additional file 3: Table S3).

Statistical analysis showed a negative correlation between sand flies captured during the surveys by LT and ST and mean, maximum and minimum RH (Spearman’s rho = -0.4 – -1, *n* = 12, *P* = 0.5167–0.0167) but the only significant negative correlation was only observed between *S. minuta* captured by LT and minimum RH (Spearman’s rho = -1, *P* = 0.0167) (Additional file 4: Table S4). Regarding temperature, sand fly captures positively correlated with mean temperature, maximum temperature and minimum temperature in both LT and ST (Spearman’s rho = 0.4–1, *n* = 12, *P* = 0.5167–0.0167) with a significant correlation only observed between *S. minuta* captured by LT and maximum temperature values (Spearman’s rho = 1, *P* = 0.0167) (Additional file 4: Table S4).

### Sand fly dissections, infection rates and molecular characterization of isolates

**Fig. 5:**
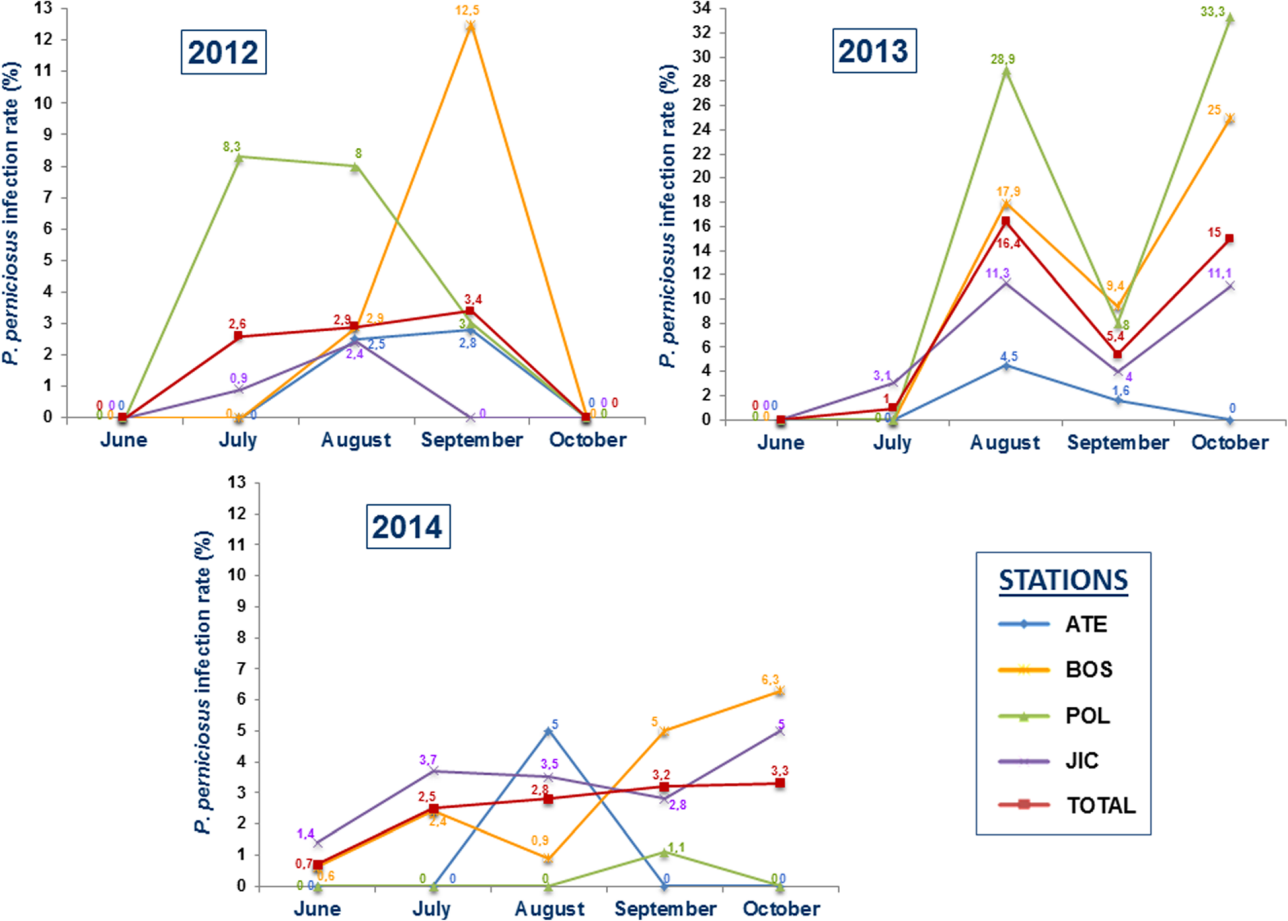
Dynamics of mean infection rates of *Phlebotomus perniciosus* by *Leishmania infantum* by station in 2012, 2013 and 2014

During the three survey periods, a total of 3203 *P. perniciosus* females collected with LT in the 4 stations of seasonal study were dissected. The global infection rate was 3.65%. In 2012, 19 out of 735 females dissected were found infected with promastigotes (2.6%). In 2013, 864 females were dissected with 57 of them infected, giving a rate of infection of 6.6%. In 2014, 1604 females were dissected and promastigotes were found in 41 specimens (2.6%). Of the dissected infected females 7.7% were gravid and 6% were semigravid. As shown in Fig. 5, infection rates dynamics fluctuated during the three periods, although 2013 was the period with highest infection rates. Specifically, an exceptional rate of infected sand flies was found by dissection in August and October 2013. Overall, sand fly dissections revealed higher parasite presence in August, September and October during the three periods. Of the isolates from dissected guts, 19.2% resulted contaminated by fungi or bacteria and in the 5.1% of the cultures the growth of the parasites was unsuccessful due to the low number of promastigotes recovered from the infected guts. A total of 67 isolates were successfully cultured and this allowed proceeding to molecular characterization. ITS1 and ITS2 sequences were obtained and analysed by comparison with GenBank database. All the isolates showed an identity of 100% with *L. infantum* strain MHOM/ES/87/Lombardi (ITS type Lombardi; AJ000295) (Additional file 5: Figure S1).

### Blood-feeding preferences and *Leishmania* detection by molecular procedures

**Fig. 6:**
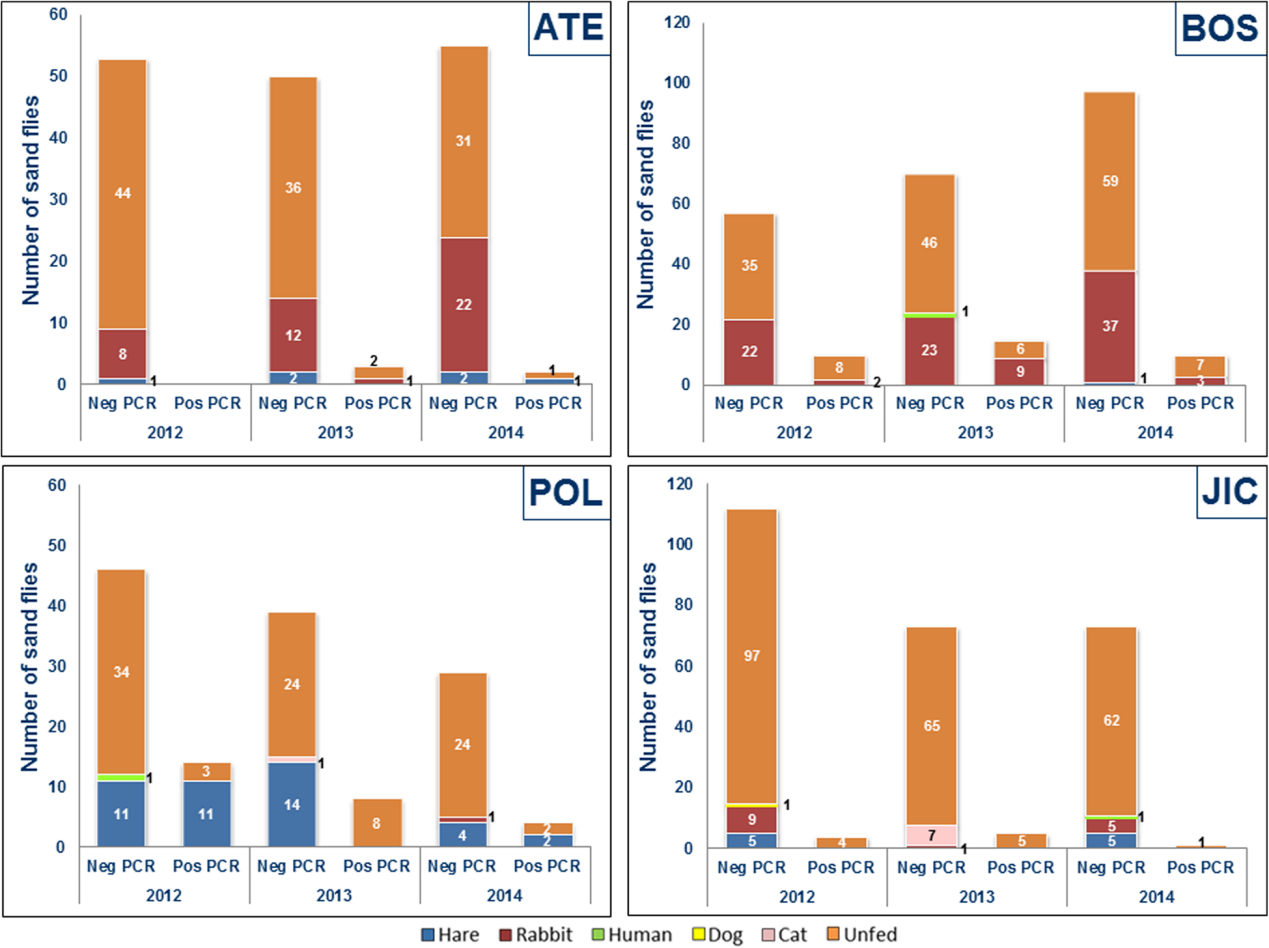
Molecular detection of *Leishmania* in fed and unfed female *Phlebotomus perniciosus* and blood meal preferences by station (ATE, BOS, POL and JIC) and survey period (2012, 2013 and 2014)

A total of 912 *P. perniciosus* females were analysed during the study, 308 of them blood-fed. Host-feeding preferences of *P. perniciosus* were investigated over the 3 years of the study, 2012 (*n* = 100), 2013 (*n* = 92) and 2014 (*n* = 116). Blood identification was achieved in 224 blood-fed *P. perniciosus* females (efficiency of 74.36%).The rate of unidentified blood meal sources in *P. perniciosus* engorged females was similar along the 3 years of the study: 29%, 23.07%, and 25% in 2012, 2013 and 2014, respectively. The following hosts have been identified: rabbit (50.33%), hare (19.16%), cat (3.57%), human (0.97%) and dog (0.33%). Differences in the identified hosts were found between the three survey periods. The percentages of females fed on rabbits were increasing during the study: 41% in 2012, 50.54% in 2013, and 58.62% in 2014. In contrast, the rate of hares as blood meal source was decreasing, 28%, 17.58% and 12.93% in 2012, 2013 and 2014, respectively. Blood preferences by station are shown in Fig. 6. It is remarkable that most of hare blood was detected in sand flies collected in the station LEG-POL and the station with more variable blood meal sources was FUE-SJIC.

Additionally the blood contained in the gut of 30.3% (10 out of 33) *S. minuta* female could be identified. All these blood meals were identified as gecko blood.

Regarding to molecular detection of *Leishmania*, a total of 41 positive blood-fed *P. perniciosus* females were found (13.31%). Three positive females were gravid (7.3%) and two were semigravid (4.87%). 2012 was the period with highest rate of infection (23%), in 2013 the rate was 11.11% and 4.76% in 2014. Sixteen (69.56%) out of 23 positive female sand flies found in 2012 were detected in POL station; 11 out of this 16 blood meal sources were identified as hare. Another two sand flies fed on rabbits were found positive in BOS station, and other 5 sand flies with unidentified blood meal source were found positive (2 in ATE and 3 in JIC stations). Ten out of 12 positive blood-fed females collected in 2013 were fed on rabbits (9 in BOS and 1 in ATE), in the other 2 flies the blood could not be identified (1 in BOS and 1 in POL). Finally, 3 out of 6 positive sand flies collected in 2014 were fed on rabbits and the other 3 on hares: 1 fed on hare in ATE, 2 fed on hares in POL and 3 fed on rabbits in BOS (Fig. 6). Infection rates of females fed on rabbits during the three collection periods were 4.97%, 21.74% and 4.41% in 2012, 2013 and 2014, respectively. As to the females fed on hares the infection rates were 39.28% in 2012, 0% in 2013 and 20% in 2014.

Concerning unfed females, 604 *P. perniciosus* were analysed for *Leishmania* detection: 225 collected in 2012; 192 in 2013 and 187 in 2014. A total of 47 females were positive for *Leishmania* (7.78%). The highest infection rate was found in 2013 (10.88%), followed by 2012 (6.69%) and 2014 (5.88%). Only one positive gravid female (2.12%) was observed. During the three survey periods, BOS was the station with higher number of infected sand flies, 21 (32.30%), followed by POL, 13 (11.71%), JIC, 10 (4.13%) and ATE, 3 (2.36%). Additionally, 40 *S. minuta* females were also investigated but no trypanosomatids were detected (Fig. 6).

## Discussion

In this work we present results on sand fly seasonal dynamics, as well as findings about sand fly infection rates and blood meal preferences in the exceptional outbreak of human leishmaniasis in an urban area of the southwestern region of Madrid (central Spain).

Anthropogenic land changes as deforestation-reforestation, road construction and urbanization seem to be a very important driver of infectious disease outbreaks giving raise to emergence or re-emergence events. These changes perturb host-parasite dynamics equilibrium in parasitic vectorial diseases, including leishmaniasis [15, 28]. The emergence of leishmaniasis in many areas can be associated with urbanization, ecological factors and climatic changes that favor the increase in vector densities and the modification of host population or its composition [29, 30]. In the case of the studied area the high densities of *P. perniciosus* found in this work could be explained because of the alteration of the periurban area which was modified in order to establish an extensive green area surrounding the different affected towns. This alteration was expected to modify hare and rabbit populations in this open space and as result, the sand fly density has probably increased [6]. This finding agrees with observations on the plasticity and adaptability to environmental changes of *P. perniciosus* observed in Italy by Tarallo [31]. Moreover, environmental changes are proved to affect sand fly population and density [32–35].

During the three survey periods four different sand fly species were found: *P. perniciosus*, *P. sergenti*, *P. papatasi* and *S. minuta*. Although *P. ariasi* have been recorded in some areas of Madrid region no specimen of this species was found in our surveys. Moreover, Tello et al. [36] only found one specimen during their collections in different sites of the affected area. We could not find any specific reason for the absence of this species, so it could be due to the homogeneity of bioclimatic and ecological variables of the study area that are unsuitable for the presence of this sand fly species [37, 38]. *Phlebotomus perniciosus* was the predominant species and *S. minuta* was the second in abundance, contrary to what has been reported elsewhere in the Madrid region [37, 38], probably because bioclimatic and environmental conditions of the area of study are more favorable for the first species. The detection of *P. sergenti* and *P. papatasi* was sporadic throughout the study probably because the ecological variables of the study area are very uniform and do not favor their presence. Sand fly density in each station showed variation between survey periods. The high growth of the rabbit population observed in BOS during the three surveys could influence the high sand fly abundance observed in this station. With the exception of this location, captures tended to decrease or maintain, possibly as a result of the control measures taken in the outbreak area such as management actions and regulation of hare and rabbit densities. Furthermore, studies carried out in France state that an area with a mean sand fly density above 20 sand flies/m^2^ is a zone with high leishmaniasis transmission [39]. According to this statement, the area studied in this work would be a zone at very high risk of transmission due to the elevated *P. perniciosus* density registered (193.60 specimens/m^2^). Differences observed between ST and LT are due to the fact that the former method captures sand flies by interception and the latter by attraction. Thus, *P. perniciosus* are more phototropic than *S. minuta*, which explains the significant RA differences between the two species in the present study as previously reported [40]. However, these results are in contrast to previous studies which recorded higher *S. minuta* density than *P. perniciosus* by using ST [36, 37]. A possible explanation could be the extraordinary *P. perniciosus* population present in the area due to a convergence of factors as an extraordinary availability of wild leporids and favorable bioclimatic conditions. Moreover, the ST were not placed in barbicans, drainage holes in walls, nor tubes; they were placed at the base of the trunks of shrubs or trees, next to deep cracks of the ground, next to wire fences, on the grates of sinkholes of sewer network, and in the proximities of the rabbit burrows [40–42].

Seasonal dynamics of *P. perniciosus* by LT showed a confluent bi-modal pattern in which the convergence of similar density peaks comprising June to September captures was observed (Fig. 3). However, a maximum peak of captures was found in August 2012, the month with lowest mean RH and highest mean temperature, which is in line with statistical analysis of correlation between captures and bioclimatic parameters. In the case of peaks of maximum number of captures observed in September 2013 and 2014 no significant correlation was observed, so other parameters such as wind, temperature and RH in the days leading up to sampling, or even the climatic conditions prevailing in the spring or winter, might have affected sand fly captures [42]. Using ST we also observed a confluent bi-modal pattern during the three periods (Fig. 4). Just like with LT, 2012 results agreed with correlation analyses; however, 2013 and 2014 surveys did not. Confluent bi-modal pattern was also described in other studies in the Mediterranean basin [42], although it is very similar to the bi-modal pattern previously reported in Spain [43–45]. In case of *S. minuta* dynamics, LT captures were not significant enough to detect a pattern. However, using ST, the maximum peak in every period was in August, the month when highest temperatures and lowest RH were recorded, except in 2014 where *S. minuta* captures remained very high in September. *Sergentomyia minuta* collections agreed with the results obtained in the correlation analysis. This kind of correlation between RH and temperature has been previously reported in entomological studies carried out in the Mediterranean region [46].

Prevalence studies of *Leishmania* infection in the phlebotomine vectors are worthwhile indicator of the intensity of *Leishmania* transmission. In the present study, we combined classical demonstration of *Leishmania* promastigotes by dissection of sand fly guts and molecular detection and identification of the parasite using PCR. Although the microscopic detection of promastigotes in the gut of dissected sand flies is a very tedious task that requires highly skilled personnel, it allows the isolation and culture of the parasite and its preservation for further studies. It also enables the incrimination of sand fly vector species in *Leishmania* foci. In contrast, molecular procedures are very accurate and less laborious than sand fly dissection. Nevertheless, the combination of these two methodologies allows an extensive and comprehensive study of infection rates and characterization of *Leishmania* parasites as reported in Italy, Turkey and Israel [47–49]. Mean infection rates found by dissection in the present work are similar to some studies carried out in Spain [4, 50–53] but higher than reported in other countries [26, 47, 48, 54]. The infection rates found in this study showed different pattern during the three surveys, possibly due to differences observed in the number of sand fly captures. Specifically, the higher infection rate found in 2013 could be linked to the increase in the human leishmaniasis cases in early 2014. In addition, the highest rates of infection were observed in months with low number of sand fly captures and by the end of the seasonal activity of sand flies, as previously reported in other studies [50, 55]. The isolates in this work were characterized as ITS-Lombardi genotype. This ITS type is identical to that reported from direct xenodiagnoses carried out in hares and rabbits from the affected area [8, 9], as well as from samples obtained from patients from the outbreak [56].

On the other hand, the detection of *Leishmania* DNA by PCR shows high values in fed (13.35%) and unfed females (7.78%). These findings disagree with data we reported before [24], possibly because of the low number of specimens analyzed in the latter study, all of them captured in October 2011 when infection rates are usually the highest. On the other hand, the results from the present study agree with other studies where infection rates of fed female sand flies are higher than in unfed [47, 57, 58]. Moreover, an elevated infection rate was found in females fed on hares, mostly in the 2012 survey. These samples represent an important number of the fed females and could influence the elevated rate of positive samples in this group. *Leishmania* detection by molecular procedures showed higher values than average rates detected by sand fly dissection, exhibiting more sensibility and accuracy. Regarding fed females, the highest rates of infection were detected in 2012 and showed a decline pattern in the following periods. This may be a result of the control measures taken in the park close to urban areas to reduce the population of hares and rabbits, potential wild reservoirs of leishmaniasis. In this way, we could verify that in 2013 and 2014 there was an elevated rabbit density in BOS, which correlates with the high sand fly infection rates found in this station. On the other hand, the drastic reduction in the number of hares in the POL station resulted in a considerably low *P. perniciosus* infection rate in 2014 (Fig. 6).

Although the leishmaniasis is considered a zoonotic disease in Spain with the dog as a main reservoir, in the focus area where this study was carried out, the prevalence of canine leishmaniasis has not undergone any increase. However lagomorphs seem to play a role as wild reservoirs of leishmaniasis in the area close the focus, as shown by direct xenodiagnoses in hares and rabbits [8, 9]. In a previous study of blood meal preferences performed in a small number of blood-fed *P. perniciosus* from the focus had already been shown that most of them were fed on rabbits and hares [9]. To find out more about host feeding preferences of sand flies collected in the park close the urban area of the focus, molecular procedures were applied. Global results of blood meal identification showed that *P. perniciosus* preferably feeds on rabbits, followed by hares, reinforcing the role these lagomorphs may be playing in the focus. Several studies on feeding habits of sand flies in *Leishmania* foci have reported an opportunistic behavior, finding engorged *P. perniciosus* females fed on different vertebrates, including humans, as well as mixed feeding sources [19, 47, 58]. In this way, a PCR restriction fragment length polymorphism methodology was optimized in order to discriminate mixed blood sources on sand flies captured in different areas of Spain [59]. Regarding the efficiency of blood-meal identification in *P. perniciosus* females, this was similar or higher to that reported by other authors using the same methodology [17, 26, 58, 60]. The results obtained in the different stations showed that in BOS rabbit was the prevalent blood meal source, possibly due to the elevated population of this mammal observed in this station during the surveys. As early as 2011, the Community of Madrid warned about the existence of an extraordinary hare population in POL. Blood meal preferences found in this station correlate with this observation. However, the number of *P. perniciosus* fed on hares has been decreasing along the surveys probably due to the different measures implemented by the regional government in the frame of the control program of the disease. The host-feeding preferences found in this work are in concordance with studies which show that these two lagomorphs are highly exposed to *P. perniciosus* bites in the focus area [10]. In addition, sand flies fed on cats were found in JIC and POL, were some colonies of these animals were observed. The variation in blood-feeding preferences observed correlates with the opportunistic feeding behavior of *P. perniciosus* (Fig. 6) [61].

We are therefore facing an area with a high *L. infantum* transmission where intensive and continuous surveillance should be taken to regulate lagomorph populations and sand fly vector densities. Information about vectors and their distribution should be updated regularly in order to manage efficient control programs of leishmaniasis in the area. Moreover, surveillance programs of leishmaniasis should be undertaken in other geographic regions endemic for leishmaniasis with similar eco-epidemiological characteristics in order to keep track of the populations of sand fly vectors, the presence of the parasite in the sand flies and the potential wild reservoirs like the leporids.

## Conclusions

Our entomological study carried out in the green park close to the urban areas of southwestern region of Madrid (Spain) affected by the outbreak of leishmaniasis revealed high densities of *P. perniciosus*, the only vector in the area. Infection rates by dissection of sand fly guts and by molecular procedures showed an important presence of *L. infantum* in the vector. From our study, it was also observed that rabbits and hares were the main blood meal sources of this sand fly species confirming the essential role of lagomorphs in sustaining the sylvatic *Leishmania* cycle in the green park.

## Additional files

Additional file 1: Table S1. Monthly relation of *P*-values of *P. perniciosus* and *S. minuta* densities through the three survey periods. Results of Kruskal-Wallis and Dunn’s multiple comparison tests. (DOCX 18 kb)

Additional file 2: Table S2. Statistical analysis of RH through the three periods. Coefficient results from Kruskal-Wallis test and *P*-values resulting from Dunn’s multiple comparison test. (DOCX 18 kb)

Additional file 3: Table S3. Statistical analysis of temperature through the three periods of the study. Coefficient results from Kruskal-Wallis test and *P*-values resulting from Dunn’s multiple comparison test. (DOCX 20 kb)

Additional file 4: Table S4. Correlation analysis between *P. perniciosus* and *S. minuta* collections and bioclimatic parameters. Statistical results. (DOCX 20 kb)

Additional file 5: Figure S1. Alignment of ITS sequences of *Leishmania infantum* isolates from 2012, 2013 and 2014 and strains with different ITS types of *L. infantum* retrieved in the Genbank: type Lombardi (AJ000295), type A (AJ634341), type B (AJ000288), type A/B (AJ634355), type E (AJ634361) and type F (AJ634370). (PDF 1192 kb)

## References

[1] World Health Organization (2010). Control of Leishmaniases.

[2] Gil-Prieto R, Walter S, Alvar J, Gil de Miguel A (2011). Epidemiology of leishmaniasis in Spain based on hospitalization records (1997–2008). Am J Trop Med Hyg.

[3] Aliaga L, Cobo F, Mediavilla JD, Bravo J, Osuna A, Amador JM, et al. Localized mucosal leishmaniasis due to *Leishmania *(*Leishmania*) *infantum*: clinical and microbiologic findings in 31 patients. Medicine. 2003;82:147–58. 10.1097/01.md.0000076009.64510.b8 12792301

[4] Rioux JA, Guilvard E, Gállego J, Moreno G, Pratlong F, Portús M, Rioux JA (1986). *Phlebotomus ariasi* Tonnoir, 1921 et *Phlebotomus perniciosus* Newstead, 1911 vecteurs du complexe *Leishmania infantum* dans un même foyer: Infestations par deux zymodèmes syntopiques. A propos d’une enquête en Catalogne (Espagne). *Leishmania*. Taxonomie et Phylogénèse. Applications écoépidémiologiques.

[5] Suárez B, Isidoro B, Santos S, Sierra MJ, Molina R, Astray J (2012). Situación epidemiológica y de los factores de riesgo de transmisión de *Leishmania infantum* en España. Rev Esp Salud Pública.

[6] Arce A, Estirado A, Ordobas M, Sevilla S, García N, Moratilla L (2013). Re-emergence of leishmaniasis in Spain: community outbreak in Madrid, Spain, 2009 to 2012. Euro Surveill.

[7] Vilas F, Carpintero J, Sevilla S, Martínez A, Ordobás M, Bernal J (2012). Brote de leishmaniasis en la zona suroeste de la Comunidad de Madrid. Medidas de investigación y control medioambiental. Profesión Veterinaria.

[8] Molina R, Jiménez MI, Cruz I, Iriso A, Martín-Martín I, Sevillano O (2012). The hare (*Lepus granatensis*) as potential sylvatic reservoir of *Leishmania infantum* in Spain. Vet Parasitol.

[9] Jiménez M, González E, Martín-Martín I, Hernández S, Molina R (2014). Could wild rabbits (*Oryctolagus cuniculus*) be reservoirs for *Leishmania infantum* in the focus of Madrid, Spain?. Vet Parasitol.

[10] Martín-Martín I, Molina R, Rohousova I, Drahota J, Volf P, Jiménez M (2014). High levels of anti-*Phlebotomus perniciosus* saliva antibodies in different vertebrate hosts from the re-emerging leishmaniosis focus in Madrid, Spain. Vet Parasitol.

[11] Moreno I, Álvarez J, García N, de la Fuente S, Martínez I, Marino E (2013). Detection of anti-*Leishmania infantum* antibodies in sylvatic lagomorphs from an epidemic area of Madrid using indirect immunofluorescence antibody test. Vet Parasitol.

[12] Ruiz-Fons F, Ferroglio E, Gortázar C (2013). *Leishmania infantum* in free-ranging hares, Spain, 2004-2010. Euro Surveill.

[13] Díaz-Sáez V, Merino-Espinosa G, Morales-Yuste M, Corpas-López V, Pratlong F, Morillas-Márquez F (2014). High rates of *Leishmania infantum* and *Trypanosoma nabiasi* infection in wild rabbits (*Oryctolagus cuninulus*) in sympatric and synthropic conditions in an endemic canine leishmaniasis area: epidemiological consequences. Vet Parasitol.

[14] García N, Moreno I, Álvarez J, de la Cruz MA, Navarro A, Pérez-Sancho M (2014). Evidence of *Leishmania infantum* infection in rabbits (*Oryctolagus cuniculus*) in a natural area of Madrid, Spain. Biomed Res Int.

[15] Ready PD (2010). Leishmaniasis emergence in Europe. Euro Surveill.

[16] Medlock JM, Hansford KM, Van Bortel W, Zeller H, Alten B (2014). A summary of the evidence for the change in European distribution of phlebotomine sand flies (Diptera: Psychodidae) of public health importance. J Vect Ecol.

[17] Branco S, Alves-Pires C, Maia C, Cortes S, Cristóvao JMS, Gonçalvez L (2012). Entomological and ecological studies in a new potencial zoonotic leishmaniasis focus in Torres Novas municipality, central region, Portugal. Acta Trop.

[18] Ballart C, Guerrero I, Castells X, Barón S, Castillejo S, Alcover MM (2014). Importance of individual analysis of environmental and climatic factors affecting the density of *Leishmania* vectors living in the same geographical area: the example of *Phlebotomus ariasi* and *P. perniciosus* in northeast Spain. Geospat Health.

[19] Maia C, Parreira R, Cristóvao JM, Freitas FB, Afonso MO, Campino L (2015). Molecular detection of *Leishmania* DNA and identification of blood meals in wild caught phlebotomine sand flies (Diptera: Psychodidae) from southern Portugal. Parasit Vectors.

[20] Bravo-Barriga D, Parreira R, Maia C, Afonso MO, Blanco-Ciudad J, Serrano FJ (2016). Detection of *Leishmania* DNA and blood meal sources in phlebotomine sand flies (Diptera: Psychodidae) in western of Spain: update on distribution and risk factors associated. Acta Trop.

[21] Gil-Collado J, Morillas-Márquez F, Sanchís-Marín MC (1989). Los flebotomos en España. Rev Sanid Hig Publica.

[22] Rioux JA, Guilvard E, Dereure J, Lanotte G, Denial M, Pratlong F, Rioux JA (1986). Infestation naturelle de *Phlebotomus papatasi* (Scopoli, 1786) par *Leishmania major* MON-25. A propos de 28 souches isolées dans un foyer du Sud marocain. *Leishmania*. Taxonomie et Phylogénèse. Applications écoépidémiologiques.

[23] Dolmatova AV, Demina NA (1971). Les phlébotomes (Phlebotominae) et les maladies qu'ils transmittent. Étude anatomique et physiologique des *Phlebotominae* ailés.

[24] Jiménez M, González E, Iriso A, Marco E, Alegret A, Fúster F (2013). Detection of *Leishmania infantum* and identification of blood meals in *Phlebotomus perniciosus* from a focus of human leishmaniasis in Madrid, Spain. Parasitol Res.

[25] Abassi I, Cunio R, Warburg A (2009). Identification of bloodmeals imbibed by phlebotomine sand flies using cytochrome *b* PCR and reverse line bloting. Vector-Borne Zoonotic Dis.

[26] Svobodová M, Alten B, Zídková L, Dvořák V, Hlavačková J, Myšková V (2009). Cutaneous leishmaniasis caused by *Leishmania infantum* transmitted by *Phlebotomus tobbi*. Int J Parasitol.

[27] Cupp EW, Zhang D, Yue X, Cupp MS, Guyer C, Sprenger TR (2004). Identification of reptilian and amphibian blood meals from mosquitoes in an eastern equine encephalomyelitis virus focus in Central Alabama. Am J Trop Med Hyg.

[28] Patz JA, Graczyk TK, Geller N, Vittor AY (2000). Effects of environmental changes on emerging parasitic diseases. Int J Parasitol.

[29] Harhay MO, Olliaro PL, Costa DL, Costa CHN (2011). Urban parasitology: visceral leishmaniasis in Brazil. Trends Parasitol.

[30] Aspöck H, Gerersdorfer T, Formayer H, Walochnik J (2008). Sandflies and sandfly-borne infections of humans in Central Europe in the light of climate change. Wien Klin Wochenschrift.

[31] Tarallo VD, Dantas-Torres F, Lia RP, Otranto D (2010). Phlebotomine sand fly population dynamics in a leishmaniasis endemic peri-urban area in southern Italy. Acta Trop.

[32] Maroli M, Feliciangeli MC, Bichaud L, Charrel RN, Gradoni L (2013). Phlebotomine sandflies and the spreading of leishmaniases and other diseases of public health concern. Med Vet Entomol.

[33] Barón SD, Morillas-Márquez F, Morales-Yuste M, Díaz-Saez V, Irigaray C, Martín-Sánchez J (2011). Risk maps for the presence and absence of *Phlebotomus perniciosus* in an endemic area of leishmaniasis in southern Spain: implications for the control of the disease. Parasitology.

[34] Fernández MS, Santini MS, Cavia R, Sandoval AE, Pérez AA, Acardi S (2013). Spatial and temporal changes in *Lutzomyia longipalpis* abundance, a *Leishmania infantum* vector in an urban area in northeastern Argentina. Mem Inst Oswaldo Cruz.

[35] Barhoumi W, Qualls WA, Archer RS, Fuller DO, Chelbi I, Cherni S (2015). Irrigation in the arid regions of Tunisia impacts the abundance and apparent density of sand fly vectors of *Leishmania infantum*. Acta Trop.

[36] Tello A, González-Mora D, Outerelo R, Iriso A, Vázquez MA (2015). The sand flies of the outbreak of leishmaniasis in south-west area of Madrid community (Diptera, Psychodidae, Phlebotominae). Bol R Soc Esp Hist Nat Sec Biol.

[37] Conesa Gallego E, Romera Lozano E, Martínez Ortega E (1999). Estudio de las poblaciones de flebotomos (Diptera, Psychodidae) de la Comunidad de Madrid (España). Anales de Biología 22 (Biología animal, 11).

[38] Gálvez R, Descalzo MA, Miró G, Jiménez MI, Martín O, Dos Santos-Brandao F, et al. Seasonal trends and spatial relations between environmental/meteorological factors and leishmaniosis sand fly vector abundances in central Spain. Acta Trop. 2010;115:95–202. 10.1016/j.actatropica.2010.02.009 20171154

[39] Rioux JA, Croset H, Lanotte G (1977). Ecologie d'un foyer Méditerranéen de leishmaniose viscérale. Essai de modélisation. Colloques lnternationaux du CNRS.

[40] Alexander B (2000). Sampling methods for phlebotomine sandflies. Med Vet Entomol.

[41] Alten B, Ozbel Y, Ergunay K, Kasap OE, Cull B, Antoniou M (2015). Sampling strategies for phlebotomine sand flies (Diptera: Psychodidae) in Europe. Bull Entomol Res.

[42] Alten B, Maia C, Afonso O, Campino L, Jiménez M, González E (2016). Seasonal dynamics of phlebotomine sand fly species proven vectors of Mediterranean leishmaniasis caused by *Leishmania infantum*. PLoS Negl Trop Dis.

[43] Morillas-Márquez F, Guevara Benítez DC, Ubeda Ontiveros JM, González CJ (1983). Fluctuations annuelles des populations de phlebotomes (Diptera, Phlebotomidae) dans la province de Grenade (Espagne). Ann Parasitol Hum Comp.

[44] Sanchís Marín MC, Morillas-Márquez F, González-Castro J, Benavides-Delgado I, Reyes MA (1986). Dinámica estacional de los flebotomos (Diptera: Psychodidae) de la provincia de Almería (España). Rev Iber Parasitol.

[45] Lucientes Curdi J, Benito de Martín MI, Castillo Hernández JA, Orcajo Teresa J (1991). Seasonal dynamics of *Larroussius* species in Aragon (N.E. Spain). Parassitologia.

[46] Prudhomme J, Rahola N, Toty C, Cassan C, Roiz D, Vergnes B (2015). Ecology and spatiotemporal dynamics of sand flies in the Mediterranean Languedoc region (Roquedur area, Gard, France). Parasit Vectors.

[47] Rossi E, Bongiorno G, Ciolli E, Di Muccio T, Scalone A, Gramiccia M (2008). Seasonal phenology, host-blood feeding preferences and natural *Leishmania* infection of *Phlebotomus perniciosus* (Diptera, Psychodidae) in a high-endemic focus of canine leishmaniasis in Rome province, Italy. Acta Trop.

[48] Kavur H, Eroglu F, Evyapan G, Demirkazik M, Alptepkin D, Koltas IS (2015). Entomological survey for sand fly fauna in Imamoglu Province (cutaneous leishmaniasis endemic region) of Adana, Turkey. J Med Entomol.

[49] Faiman R, Abbasi I, Jaffe C, Motro Y, Nasereddin A, Schnur LF (2013). A newly emerged cutaneous leishmaniasis focus in northern Israel and two new reservoir hosts of *Leishmania major*. PLoS Negl Trop Dis.

[50] Morillas F, Sanchíz-Marín MC, Martín-Sánchez J, Acedo SC (1991). On *Phlebotomus perniciosus* Newstead, 1911 (Diptera, Phlebotomidae) in the province of Almería in southeastern Spain. Parassitologia.

[51] Gallego M, De Colmenares M, Castillejos S, Valls D, Riera C, Fisa R (1993). Estudio del parasitismo y de la edad fisiológica de los flebotomos del Priorato.

[52] Morillas F, Sanchez Rabasco F, Ocaña J, Martin-Sanchez J, Ocaña-Wihelmi J, Acedo C (1996). Leishmaniosis in the focus of the Axarquía region, Malaga province, southern Spain: a survey of the human, dog, and vector. Parasitol Res.

[53] Lucientes Curdi J, Sánchez-Acedo C, Castillo-Hernández JA, Estrada-Peña A (1998). Sobre la infección natural por *Leishmania* en *Phlebotomus perniciosus* Newstead, 1911 y *Phlebotomus ariasi* Tonnoir, 1921, en el foco de leishmaniosis de Zaragoza. Rev Iber Parasitol.

[54] Yaghoobi-Ershadi MR, Marvi-Moghadam N, Jafari R, Akhavan AA, Solimani H, Zahrai-Ramazani AR (2015). Some epidemiological aspects of cutaneous leishmaniasis in a new focus, Central Iran. Dermatol Res Pract.

[55] Killick-Kendrick R, Rioux JA (2002). Mark-release-recapture sand flies fed on leishmania dogs: the natural life-cycle of *Leishmania infantum* in *Phlebotomus ariasi*. Parassitologia.

[56] Chicharro C, Llanes-Acevedo IP, García E, Nieto J, Moreno J, Cruz I (2013). Molecular typing of *Leishmania infantum* isolates from a leishmaniasis outbreak in Madrid, Spain, 2009 to 2012. Euro Surveill.

[57] Tiwary P, Kumar D, Mishra M, Singh RP, Rai M, Sundar S (2013). Seasonal variation in the prevalence of sand flies infected with *Leishmania donovani*. PLoS One.

[58] Es-Sette N, Ajaoud M, Laamrani-Idrissi A, Mellouki F, Lemrani M (2014). Molecular detection and identification of *Leishmania* infection in naturally infected sand flies in a focus of cutaneous leishmaniasis in northern Morocco. Parasit Vectors.

[59] González E, Gállego M, Molina R, Abras A, Alcover MM, Ballart C (2015). Identification of blood meals in field captured sand flies by a PCR-RFLP approach based on cytochrome *b* gene. Acta Trop.

[60] Paternina LE, Verbel-Vergara D, Romero-Ricardo L, Pérez-Doria A, Paternina-Gómez M, Martínez L (2016). Evidence for anthropophily in five species of phlebotomine sand flies (Diptera: Psychodidae) from northern Colombia, revealed by molecular identification bloodmeals. Acta Trop.

[61] Bongiorno G, Habluetzel A, Khoury C, Maroli M (2003). Host preferences of phlebotomine sand flies at a hypoendemic focus of canine leishmaniasis in central Italy. Acta Trop.

