# Supplementary material for: Phlebotomine sand fly survey in the focus of leishmaniasis in Madrid, Spain (2012–2014): seasonal dynamics, Leishmania infantum infection rates and blood meal preferences
Source: Parasit Vectors. 2017 Aug 1;10:368. doi: 10.1186/s13071-017-2309-z (PMC5540423; doi:10.1186/s13071-017-2309-z)
Supplement: Supplementary file 1 — Monthly relation of P-values of P. perniciosus and S. minuta densities through the three survey periods. Results of Kruskal-Wallis and Dunn’s multiple comparison tests. (DOCX 18 kb) [file 13071_2017_2309_MOESM1_ESM.docx]

**S1. Table.** Monthly relation of *p*-values of *P. perniciosus* and *S. minuta* densities through the three survey periods. Results of Kruskal-Wallis test and Dunn’s multiple comparison test

|  | *P. perniciosus* | *S. minuta* |
| --- | --- | --- |
| June | 0.0048* | 0.8906 |
| July | 0.6667 | 0.7061 |
| August | 0.0132* | 0.9945 |
| September | 0.1219 | 0.8151 |
| October | 0.0002* | 0.2397 |

|  | June | | July | | August | | September | | October | |
| --- | --- | --- | --- | --- | --- | --- | --- | --- | --- | --- |
| Years | ***P. perniciosus*** | ***S. minuta*** | ***P. perniciosus*** | ***S. minuta*** | ***P. perniciosus*** | ***S. minuta*** | ***P. perniciosus*** | ***S. minuta*** | ***P. perniciosus*** | ***S. minuta*** |
| 2012 *vs.* 2013 | 0.0182* | > 0.9999 | 0.9805 | > 0.9999 | 0.0725 | > 0.9999 | 0.1186 | > 0.9999 | 0.3502 | > 0.9999 |
| 2012 *vs.* 2014 | > 0.9999 | > 0.9999 | > 0.9999 | > 0.9999 | > 0.9999 | > 0.9999 | > 0.9999 | > 0.9999 | 0.0051* | 0.257 |
| 2013 *vs.* 2014 | 0.1497 | > 0.9999 | > 0.9999 | > 0.9999 | 0.0428* | > 0.9999 | 0.7181 | > 0.9999 | 0.3502 | > 0.9999 |

*Significant values (*p* ≤ 0.05)
