# Supplementary material for: Phlebotomine sand fly survey in the focus of leishmaniasis in Madrid, Spain (2012–2014): seasonal dynamics, Leishmania infantum infection rates and blood meal preferences
Source: Parasit Vectors. 2017 Aug 1;10:368. doi: 10.1186/s13071-017-2309-z (PMC5540423; doi:10.1186/s13071-017-2309-z)
Supplement: Supplementary file 2 — Statistical analysis of RH through the three periods. Coefficient results from Kruskal-Wallis test and P-values resulting from Dunn’s multiple comparison test. (DOCX 18 kb) [file 13071_2017_2309_MOESM2_ESM.docx]

|  | Mean RH | Max. RH | Min. RH |
| --- | --- | --- | --- |
| June | 0.0286* | 0.3282 | 0.1304 |
| July | 0.0667 | 0.0004* | 0.0398* |
| August | 0.5333 | 0.0002* | 0.0002* |
| September | 0.9333 | 0.4933 | 0.1942 |
| October | 0.4 | 0.0519 | 0.0002* |

**S2. Table**. Statistical analysis of RH through the three periods. Coefficient results from Kruskal-Wallis test and *p*-values resulting from Dunn’s multiple comparison test

|  | July | | | August | | | September | | | October | | |
| --- | --- | --- | --- | --- | --- | --- | --- | --- | --- | --- | --- | --- |
| Years | **Mean RH** | **Max RH** | **Min RH** | **Mean RH** | **Max RH** | **Min RH** | **Mean RH** | **Max RH** | **Min RH** | **Mean RH** | **Max RH** | **Min RH** |
| 2012 *vs.* 2013 | 0.0977 | 0.0003* | 0.0361 | 0.5445 | 0.0442* | 0.0217* | > 0.9999 | > 0.9999 | > 0.9999 | 0.3266 | 0.198 | 0.0195* |
| 2012 *vs.* 2014 | 0.8553 | 0.0589 | > 0.9999 | 0.8553 | 0.0001* | 0.4718 | > 0.9999 | > 0.9999 | 0.6086 | > 0.9999 | 0.0647 | 0.0001* |
| 2013 *vs.* 2014 | 0.8553 | 0.3348 | 0.3462 | > 0.9999 | 0.2692 | 0.0001* | > 0.9999 | 0.708 | 0.2399 | > 0.9999 | > 0.9999 | 0.5372 |

*Significant values (*p*-value≤0.05)
