# Supplementary material for: Phlebotomine sand fly survey in the focus of leishmaniasis in Madrid, Spain (2012–2014): seasonal dynamics, Leishmania infantum infection rates and blood meal preferences
Source: Parasit Vectors. 2017 Aug 1;10:368. doi: 10.1186/s13071-017-2309-z (PMC5540423; doi:10.1186/s13071-017-2309-z)
Supplement: Supplementary file 3 — Statistical analysis of temperature through the three periods of the study. Coefficient results from Kruskal-Wallis test and P-values resulting from Dunn’s multiple comparison test. (DOCX 20 kb) [file 13071_2017_2309_MOESM3_ESM.docx]

**S3. Table.** Statistical analysis of temperature through the three periods of the study. Coefficient results from Kruskal-Wallis test and *p*-values resulting from Dunn’s multiple comparison test.

|  | Mean Tª | Max. Tª | Min. Tª |
| --- | --- | --- | --- |
| June | 0.0667 | 0.0002* | 0.0002* |
| July | 0.2 | 0.0468* | 0.0358* |
| August | 0.0667 | 0.0007* | 0.0031* |
| September | 0.3333 | 0.0621 | 0.0063* |
| October | 0.0667 | 0.0003* | 0.0001* |

|  | July | | | August | | | September | | | October | | |
| --- | --- | --- | --- | --- | --- | --- | --- | --- | --- | --- | --- | --- |
| Years | **Mean Tª** | **Max Tª** | **Min Tª** | **Mean Tª** | **Max Tª** | **Min Tª** | **Mean Tª** | **Max Tª** | **Min Tª** | **Mean Tª** | **Max Tª** | **Min Tª** |
| 2012 vs. 2013 | 0.1843 | > 0.9999 | 0.031* | 0.8553 | 0.0016* | 0.0217* | 0.3266 | > 0.9999 | 0.0053* | 0.8553 | 0.0003* | 0.0253* |
| 2012 vs. 2014 | > 0.9999 | 0.2395 | 0.8898 | 0.0977 | 0.0055* | 0.4718 | > 0.9999 | 0.4546 | 0.8661 | 0.0977 | 0.0111* | < 0.0001* |
| 2013 vs. 2014 | 0.5445 | 0.0508 | 0.3845 | 0.8553 | > 0.9999 | 0.0001* | 0.3266 | 0.0584 | 0.1157 | 0.8553 | 0.9642 | 0.3848 |

*significant values (*p*-value≤0.05)
