# Supplementary material for: Phlebotomine sand fly survey in the focus of leishmaniasis in Madrid, Spain (2012–2014): seasonal dynamics, Leishmania infantum infection rates and blood meal preferences
Source: Parasit Vectors. 2017 Aug 1;10:368. doi: 10.1186/s13071-017-2309-z (PMC5540423; doi:10.1186/s13071-017-2309-z)
Supplement: Supplementary file 4 — Correlation analysis between P. perniciosus and S. minuta collections and bioclimatic parameters. Statistical results. (DOCX 20 kb) [file 13071_2017_2309_MOESM4_ESM.docx]

|  | **Spearman coefficient** | ***p*-value** |
| --- | --- | --- |
| **Light traps** |  |  |
| *P. perniciosus* x mean RH | - 0.7 | 0.2333 |
| *P. perniciosus* x max. RH | - 0.7 | 0.2333 |
| *P. perniciosus* x min. RH | - 0.9 | 0.0833 |
| *S. minuta* x mean RH | - 0.9 | 0.0833 |
| *S. minuta* x max. RH | - 0.9 | 0.0833 |
| *S. minuta* x min. RH | - 1 | 0.0167* |
|  |  |  |
| **Sticky traps** |  |  |
| *P. perniciosus* x mean RH | - 0.4 | 0.5167 |
| *P. perniciosus* x max. RH | - 0.6 | 0.3500 |
| *P. perniciosus* x min. RH | - 0.7 | 0.2333 |
| *S. minuta* x mean RH | - 0.7 | 0.2333 |
| *S. minuta* x max. RH | - 0.7 | 0.2333 |
| *S. minuta* x min. RH | - 0.9 | 0.0833 |

|  | **Spearman coefficient** | ***p*-value** |
| --- | --- | --- |
| **Light traps** |  |  |
| *P. perniciosus* x mean Tª | 0.7 | 0.2333 |
| *P. perniciosus* x max. Tª | 0.9 | 0.0833 |
| *P. perniciosus* x min. Tª | 0.7 | 0.2333 |
| *S. minuta* x mean Tª | 0.9 | 0.0833 |
| *S. minuta* x max. Tª | 1 | 0.0167* |
| *S. minuta* x min. Tª | 0.9 | 0.0833 |
|  |  |  |
| **Sticky traps** |  |  |
| *P. perniciosus* x mean Tª | 0.4 | 0.5167 |
| *P. perniciosus* x max. Tª | 0.7 | 0.2333 |
| *P. perniciosus* x min. Tª | 0.6 | 0.3500 |
| *S. minuta* x mean Tª | 0.7 | 0.2333 |
| *S. minuta* x max. Tª | 0.9 | 0.0833 |
| *S. minuta* x min. Tª | 0.7 | 0.2333 |

**S4. Table.** Correlation analysis between *P. perniciosus* and *S. minuta* collections and bioclimatic parameters. Statistical results.

*significant values (*p*-value≤0.05)
