# Supplementary figures and images for: Phlebotomine sand fly survey in the focus of leishmaniasis in Madrid, Spain (2012–2014): seasonal dynamics, Leishmania infantum infection rates and blood meal preferences
Source: Parasit Vectors. 2017 Aug 1;10:368. doi: 10.1186/s13071-017-2309-z (PMC5540423; doi:10.1186/s13071-017-2309-z)

Alignment of isolates from 2012

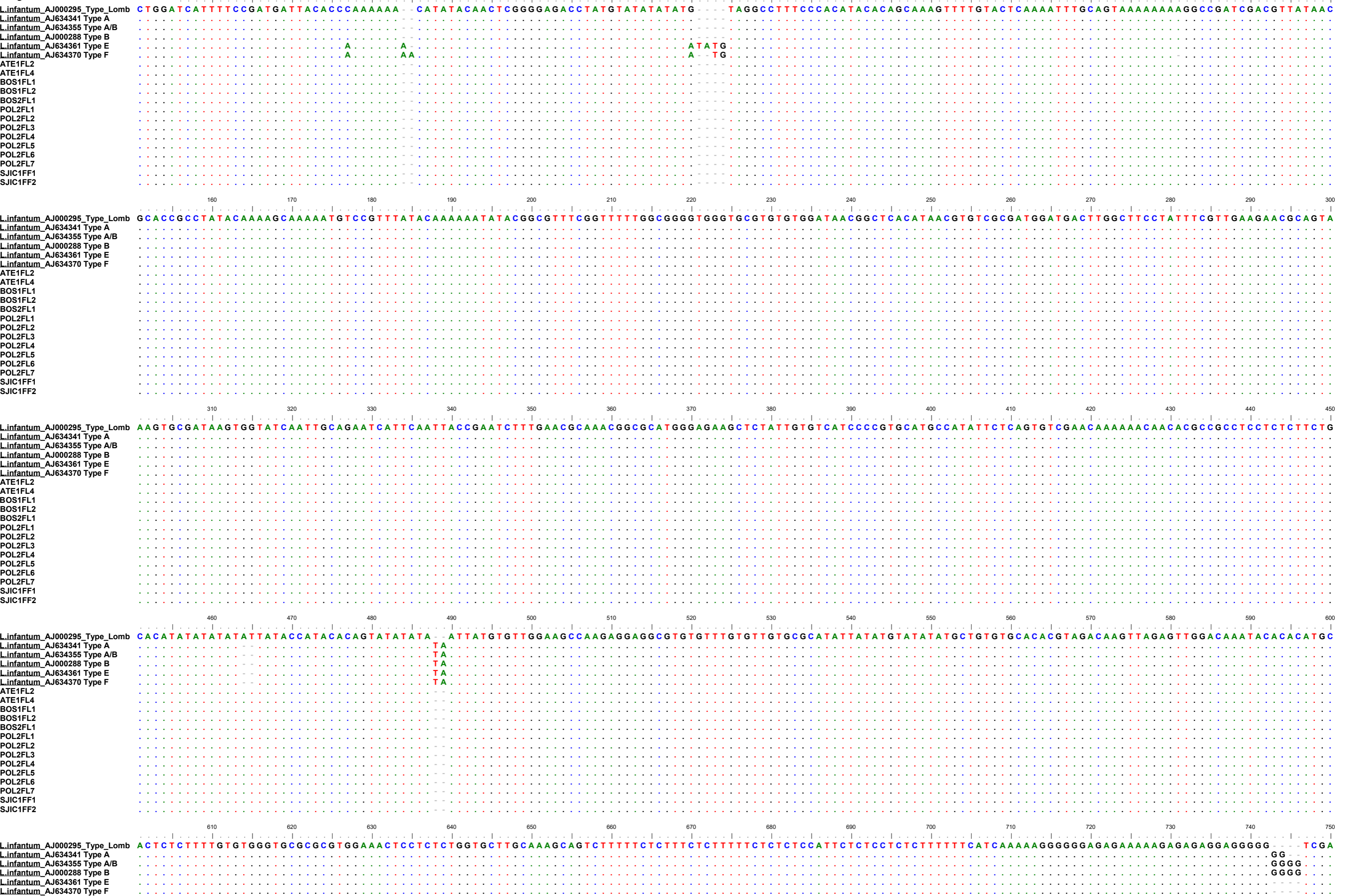

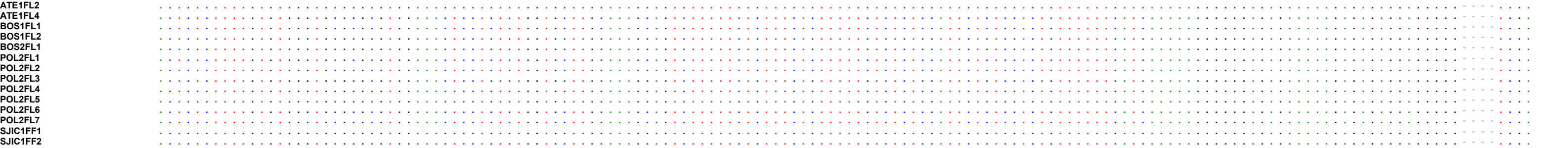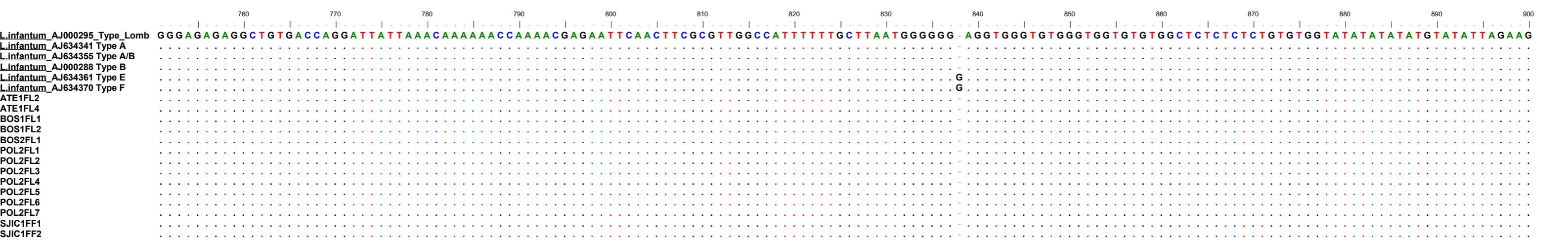

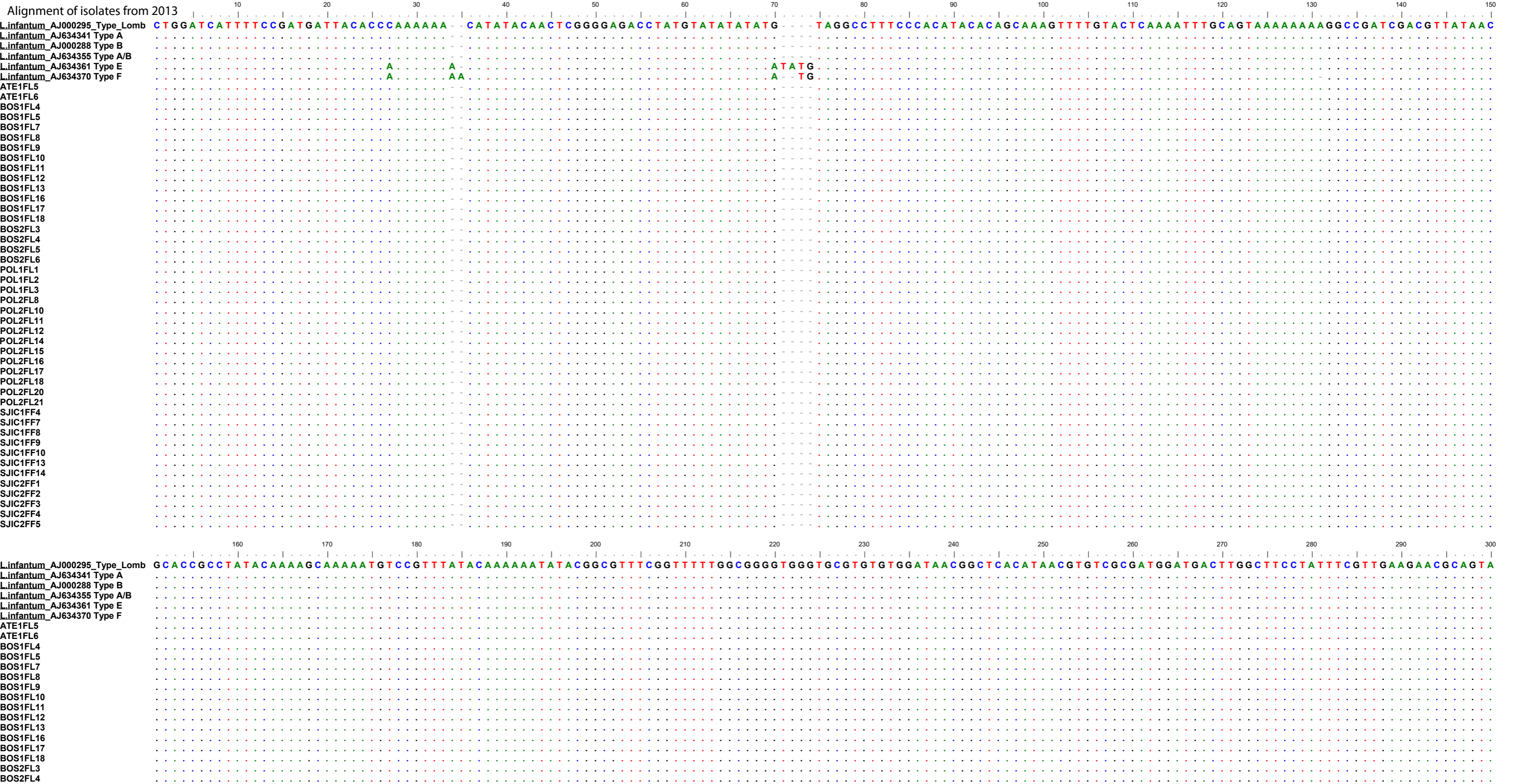

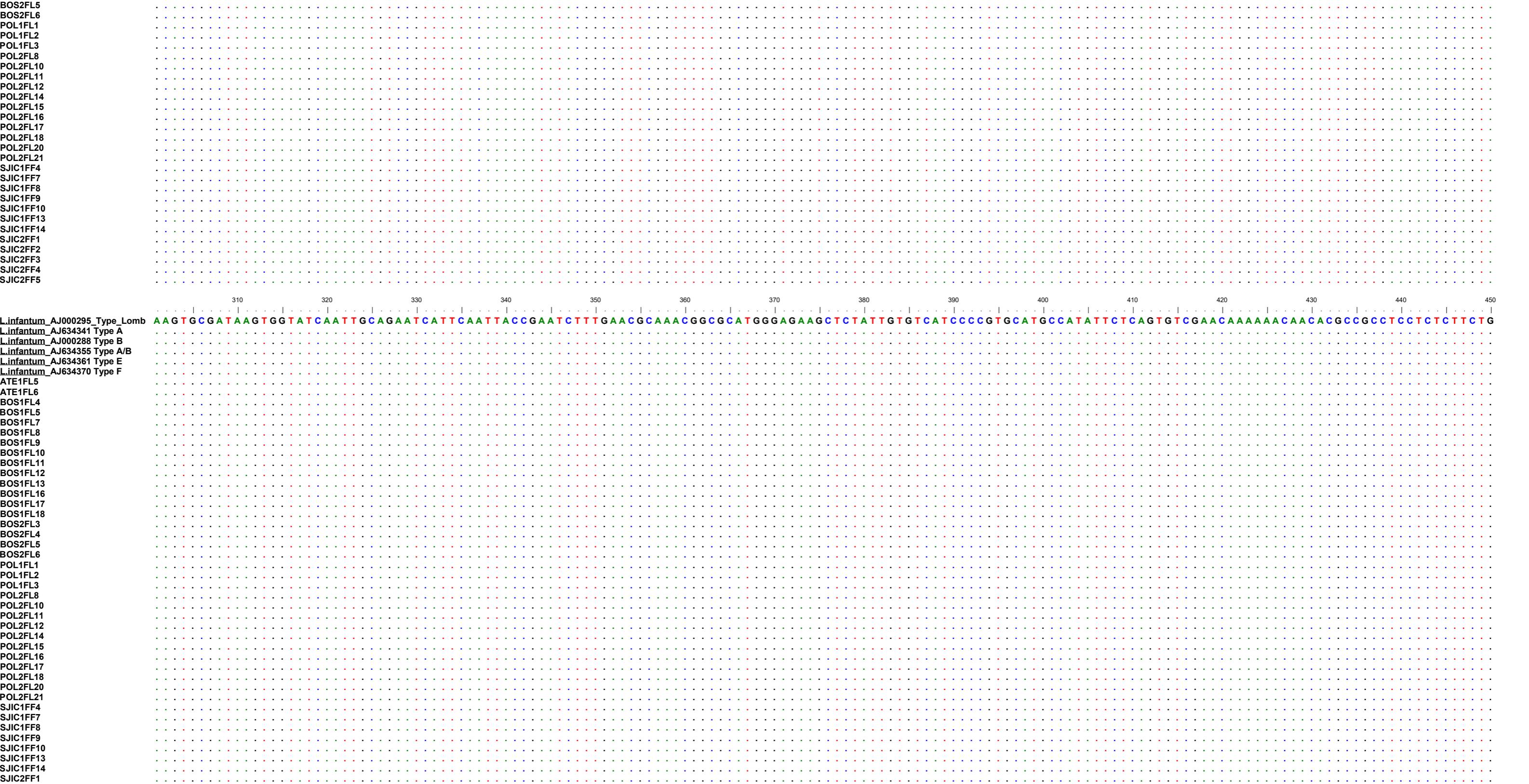

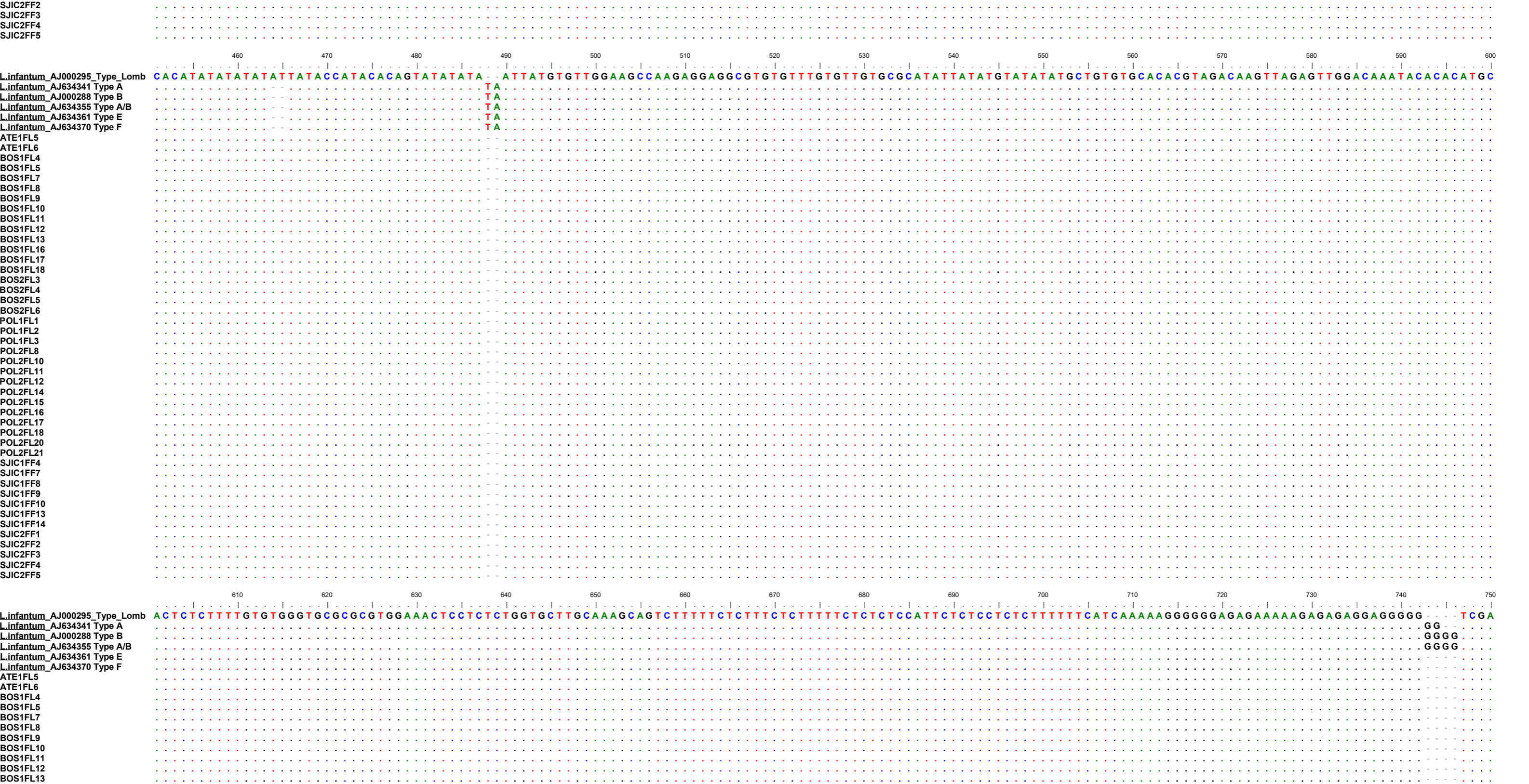

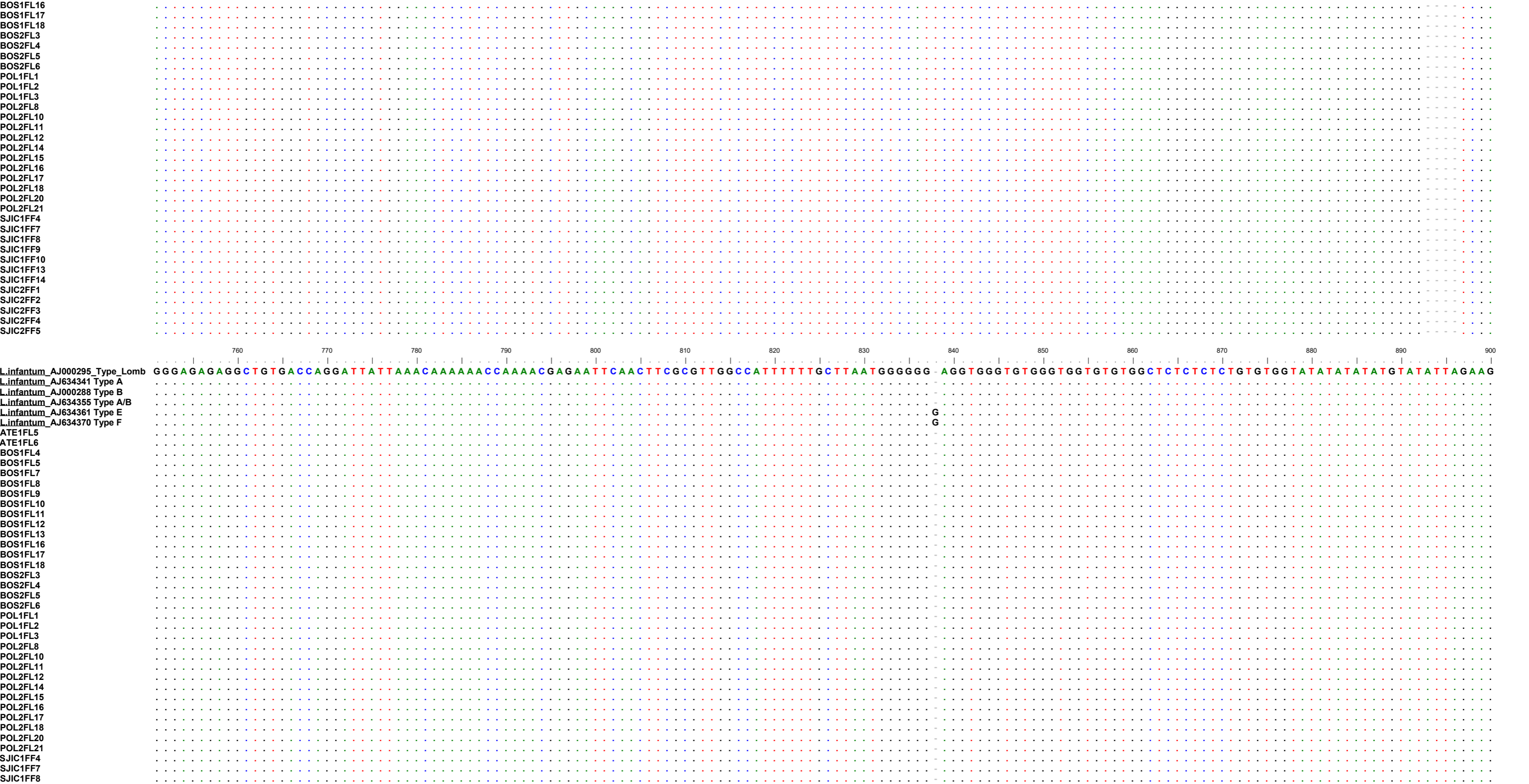

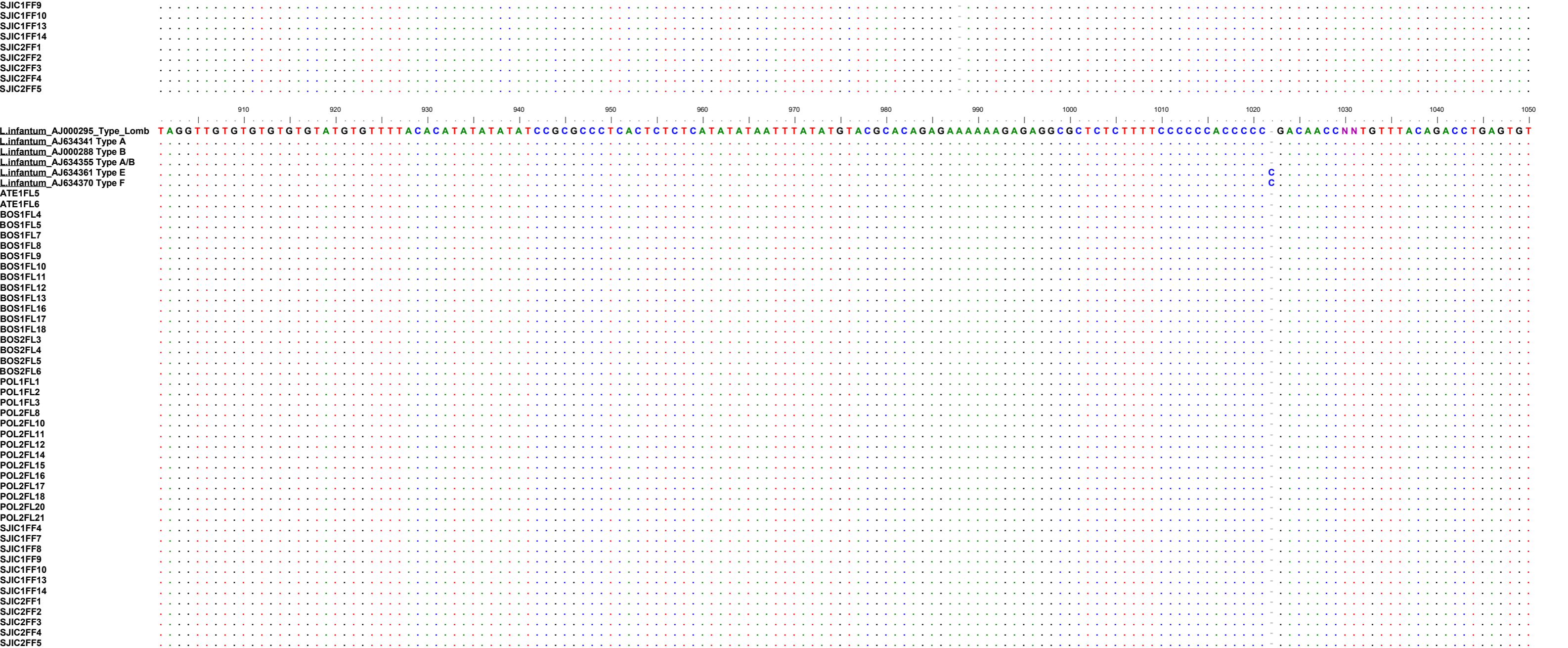

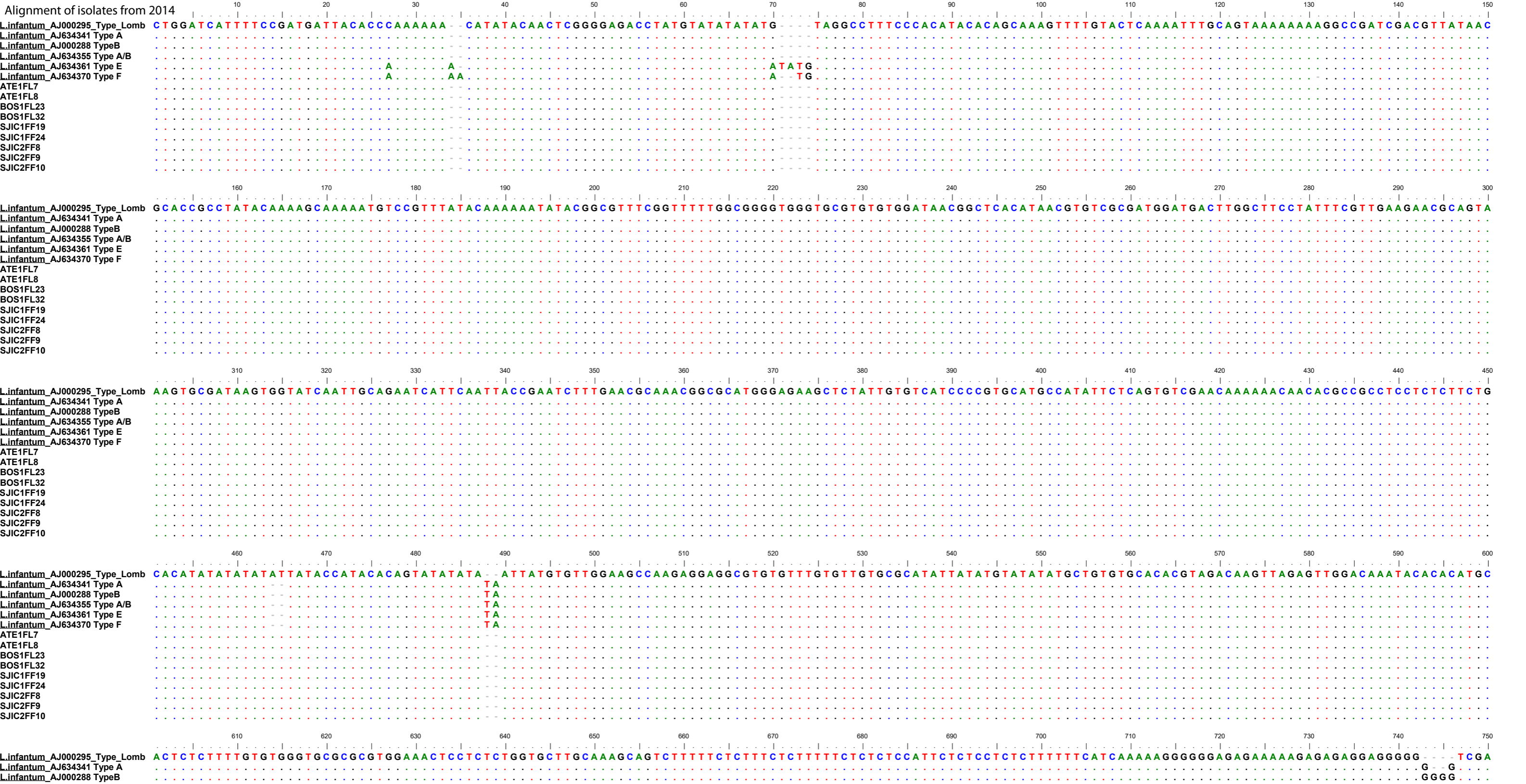

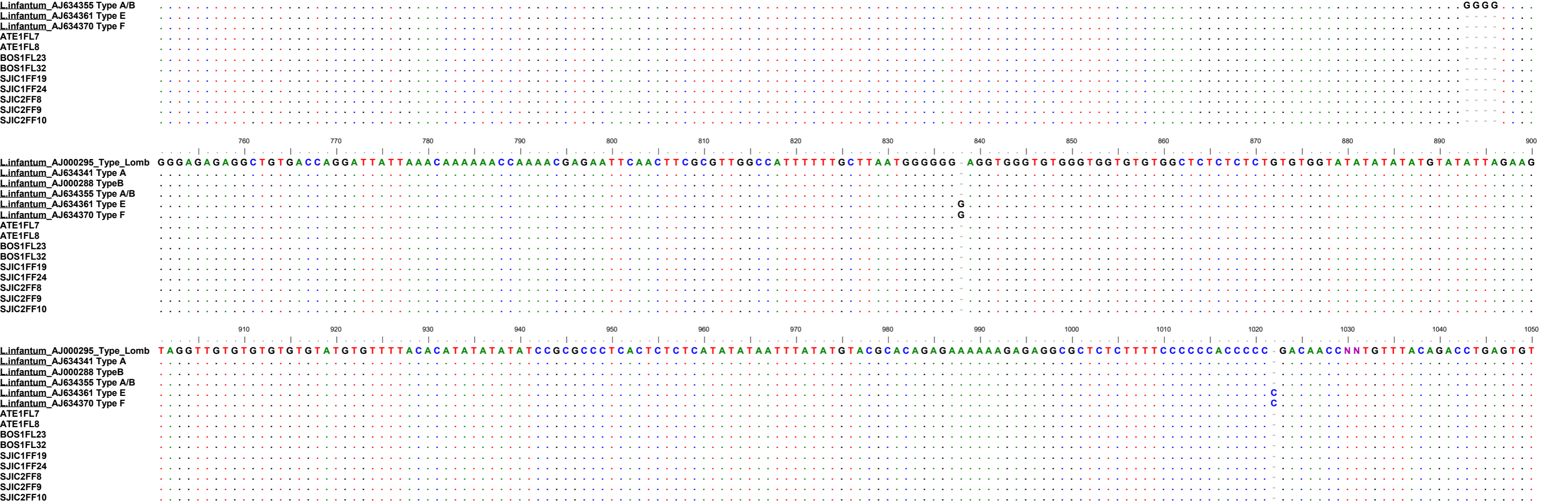

Supplement: Supplementary file 5 — Alignment of ITS sequences of Leishmania infantum isolates from 2012, 2013 and 2014 and strains with different ITS types of L. infantum retrieved in the Genbank: type Lombardi (AJ000295), type A (AJ634341), type B (AJ000288), type A/B (AJ634355), type E (AJ634361) and type F (AJ634370). (PDF 1192 kb) [file 13071_2017_2309_MOESM5_ESM.pdf]
